# Calmodulin fishing with a structurally disordered bait triggers CyaA catalysis

**DOI:** 10.1371/journal.pbio.2004486

**Published:** 2017-12-29

**Authors:** Darragh P. O’Brien, Dominique Durand, Alexis Voegele, Véronique Hourdel, Marilyne Davi, Julia Chamot-Rooke, Patrice Vachette, Sébastien Brier, Daniel Ladant, Alexandre Chenal

**Affiliations:** 1 Institut Pasteur, UMR CNRS 3528, Chemistry and Structural Biology Department, Paris, France; 2 Institute for Integrative Biology of the Cell (I2BC), CEA, CNRS, Univ. Paris-Sud, Université Paris-Saclay, Gif-sur-Yvette cedex, France; 3 Institut Pasteur, USR CNRS 2000, Chemistry and Structural Biology Department, CITECH, Paris, France; UMDNJ/Robert Wood Johnson Medical School, United States of America

## Abstract

Once translocated into the cytosol of target cells, the catalytic domain (AC) of the adenylate cyclase toxin (CyaA), a major virulence factor of *Bordetella pertussis*, is potently activated by binding calmodulin (CaM) to produce supraphysiological levels of cAMP, inducing cell death. Using a combination of small-angle X-ray scattering (SAXS), hydrogen/deuterium exchange mass spectrometry (HDX-MS), and synchrotron radiation circular dichroism (SR-CD), we show that, in the absence of CaM, AC exhibits significant structural disorder, and a 75-residue-long stretch within AC undergoes a disorder-to-order transition upon CaM binding. Beyond this local folding, CaM binding induces long-range allosteric effects that stabilize the distant catalytic site, whilst preserving catalytic loop flexibility. We propose that the high enzymatic activity of AC is due to a tight balance between the CaM-induced decrease of structural flexibility around the catalytic site and the preservation of catalytic loop flexibility, allowing for fast substrate binding and product release. The CaM-induced dampening of AC conformational disorder is likely relevant to other CaM-activated enzymes.

## Author summary

Calmodulin is a widespread and highly conserved protein that interacts with a wide variety of eukaryotic proteins and enzymes, controlling their activities in response to calcium. The adenylate cyclase toxin (CyaA) of *Bordetella pertussis*, the causative agent of whooping cough, is one such calmodulin target. Once transported across the plasma membrane of eukaryotic cells, the catalytic domain (AC) of CyaA is activated by calmodulin, producing high levels of cAMP, which can induce cell death. We use an integrative structural biology approach combining several biophysical techniques to characterize the structural rearrangements in AC upon calmodulin binding and to elucidate their relationship to CyaA activation. We show that a disordered stretch of 75 amino acid residues in AC serves as a bait for calmodulin capture. Binding induces significant folding within this region, a prerequisite for CyaA activation. Calmodulin binding promotes the stabilization of the distant catalytic site, whilst maintaining its catalytic loop in a flexible and exposed state. Both phenomena contribute to the high enzymatic activity of AC, allowing for fast substrate binding and cAMP release. The calmodulin-induced reduction of AC conformational disorder is likely relevant to other calmodulin-activated enzymes.

## Introduction

Calmodulin (CaM) is a major mediator of calcium signaling in eukaryotic cells, ubiquitously distributed and highly conserved in the whole eukaryotic kingdom. This small acidic protein of 148 amino acids interacts with a wide variety of target proteins or enzymes to control their activities in response to calcium. CaM is made of two pairs of calcium-binding EF-hand motifs that are connected by a long flexible helix, adopting a dumbbell shape in solution. The binding of calcium ions to each EF hand triggers conformational changes that result in the exposure of hydrophobic patches, altering the association of CaM with its target proteins.

Typically, CaM interacts with its targets by binding a short segment, or CaM-binding site (CBS), of about 20–30 residues that are positively charged and can adopt an amphipathic helical structure. CaM binding to the CBS is associated with a large conformational change as the protein bends from its extended dumbbell shape into a more globular structure in which its two N- and C-lobes wrap around the target helical sequence. In many instances (e.g., myosin light chain kinase [MLCK], CaM kinases, calcineurin, CaM-activated Ca-ATPase/pumps), the CBS sequence is proximal in the primary structure to an auto-inhibitory domain (AID), which occupies the catalytic site of the enzyme in the resting state, thus inhibiting enzymatic activity. Binding of CaM to the CBS induces a conformational change that displaces the AID from the enzymatic site to release the full catalytic activity.

An original mechanism of CaM regulation has been uncovered in two toxins produced by two pathogenic bacteria, *Bacillus anthracis*, the causative agent of anthrax, and *B*. *pertussis*, responsible for whooping cough. These toxins, the edema factor and the adenylate cyclase toxin (CyaA), respectively, are adenylate cyclases secreted by the bacteria and are able to invade eukaryotic target cells, in which they are potently activated by CaM to produce cytotoxic, supraphysiological levels of cAMP [1, 2]. CyaA is essential for the early steps of colonization of the respiratory tract by *B*. *pertussis*, selectively targeting the innate immune cells to favor bacterial survival. CyaA is a 1,706-residue-long bifunctional protein; the CaM-activated, catalytic domain (AC) is located in the 364 amino-proximal residues, while the carboxy-terminal 1,342 residues are involved in toxin binding to target cells and delivery of the AC domain across the plasma membrane to the cell cytosol [3–5].

How CaM binding regulates the activity of the edema factor toxin has been illuminated by a series of structural studies carried out by W.J. Tang and colleagues, who solved the structures of both the CaM-free and -bound forms [6]. Edema factor consists of an N-terminal protective antigen (PA)-binding domain; a catalytic core made up of two globular subdomains, C_A_ and C_B_; and a C-terminal helical domain. The latter makes substantial contacts with the catalytic core in the absence of CaM and locks the enzyme in an inactive state. Most of these contacts are disrupted upon CaM binding, which inserts in an extended conformation between the catalytic core and the helical domain. Through a series of conformational changes, CaM indirectly stabilizes the conformation of a substrate-binding loop that is fully disordered in the CaM-free form.

In contrast to edema factor, the exact mechanism of CyaA activation by CaM is unclear. To date, the structures of AC in the absence of CaM and of AC bound to full-length CaM remain unsolved; Guo and colleagues [7] have determined the structure of AC in complex with the C-terminal domain of CaM (C-CaM) only. Although AC and the edema factor display limited sequence identity, their catalytic cores share substantial structural similarity: like edema factor, AC is organized into two globular subdomains, C_A_ and C_B_. The catalytic site, located at the interface between these two domains, is essentially identical to that of the edema factor: it is made of three highly conserved regions (catalytic region 1 [CR1], residues 54–77; catalytic region 2 [CR2], residues 184–198; and catalytic region 3 [CR3], residues 295–315) that are directly involved in substrate binding and catalysis. Nevertheless, the edema factor and AC significantly differ in CaM binding. AC has no equivalent of the edema factor C-terminal helical domain that is involved in CaM binding and activation. Instead, C-CaM binds mainly to the H-helix (spanning residues 234–254 [7]), with typical characteristics of classical CBS, i.e., a basic and amphipathic sequence, which protrudes from the C_A_ subdomain. C-CaM also interacts with a loop-helix-loop (LHL) motif (residues 341–364) located at the very C-terminal end of AC. As this segment directly contacts the CR3 catalytic loop, C-CaM could thus indirectly stabilize it in a configuration favorable for catalysis [7, 8].

Herein, we have characterized the conformational changes of AC upon binding to its CaM activator through a combination of structural studies in solution, including synchrotron radiation circular dichroism (SR-CD), hydrogen/deuterium exchange mass spectrometry (HDX-MS), and small-angle X-ray scattering (SAXS). Our results indicate that, in the absence of CaM, AC exhibits a large intrinsically disordered region (IDR) of about 75 residues (circa 20% of the AC domain) encompassing the main CBS. CaM binding triggers the folding of the CBS and a strong overall reduction in disorder that is likely crucial for AC activation. The presence of a large IDR in AC may facilitate intoxication (translocation across the membrane) of target cells and subsequently favor its association with the CaM activator via a “fly-casting” mechanism [9].

## Results

We performed parallel studies of five different samples using both HDX-MS and SAXS: CaM and AC alone, CaM in complex with the H-helix peptide from AC, and a peptide from myosin light-chain kinase (MLCK) as a reference, and finally, the AC-CaM complex. SAXS measurements were recorded using the size exclusion chromatography followed by SAXS (SEC-SAXS) setup available on the SWING beamline at the SOLEIL synchrotron (a succinct description of instrumental conditions is given in Materials and methods and [10]). All SAXS experimental and analytical details are given in S1 Table, together with the values of global structural parameters (radius of gyration [R_g_], maximal dimension [D_max_], and molecular mass) derived from the scattering data. Of note, all molecular mass values indicate that the investigated particles are present as monomers of AC or CaM in solution. HDX-MS experimental details are to be found in the relevant section of Materials and methods.

### Structural model of the isolated AC domain

**Fig 1:**
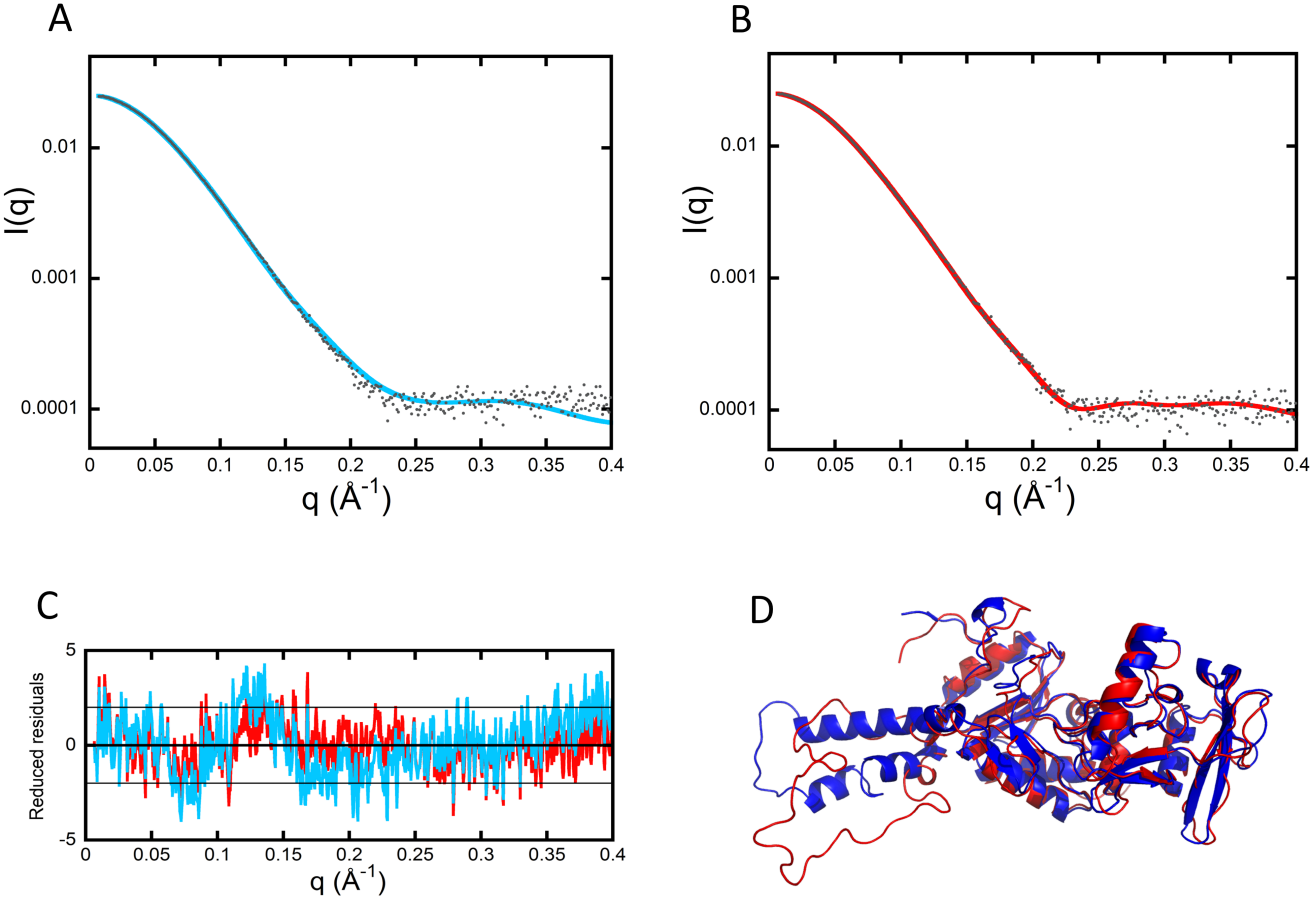
SAXS models of isolated AC. (A) Adjustment of the curve calculated from the crystal structure of AC extracted from the pdb dataset 1YRU (blue curve) against experimental data (black dots). (B) Adjustment obtained by releasing the residues comprising helices F through H′ (red curve). (C) Distribution of reduced residuals corresponding to the two fits shown in panels (A) and (B), using the same color code. (D) Crystal structure (blue) and conformation with relaxed F–H′-helices (red). AC, adenylate cyclase catalytic domain; pdb, Protein Data Bank; SAXS, small-angle X-ray scattering.

SAXS data from the free AC protein were initially modeled, starting from the atomic coordinates of AC extracted from the Protein Data Bank (pdb) dataset 1YRU of the crystal structure of AC in complex with the C-CaM [7]. Using AllosModFoxs [11], we adjusted the calculated scattering profile against experimental SAXS data by refining the proposed position of the missing residues in 1YRU (residues 226–232, defined as the Hom-loop, and the first six N-terminal residues), while keeping the AC structure unchanged. An example of one of the fits (χ^2^ = 2.57) obtained in this way is shown in Fig 1A. The corresponding model, shown in blue in Fig 1D, suggests that AC adopts a conformation roughly similar to that of the crystal, albeit with significant differences. Using the same degrees of freedom and a description in terms of ensemble of conformations did not allow us to further improve the agreement with our experimental data.

**Fig 2:**
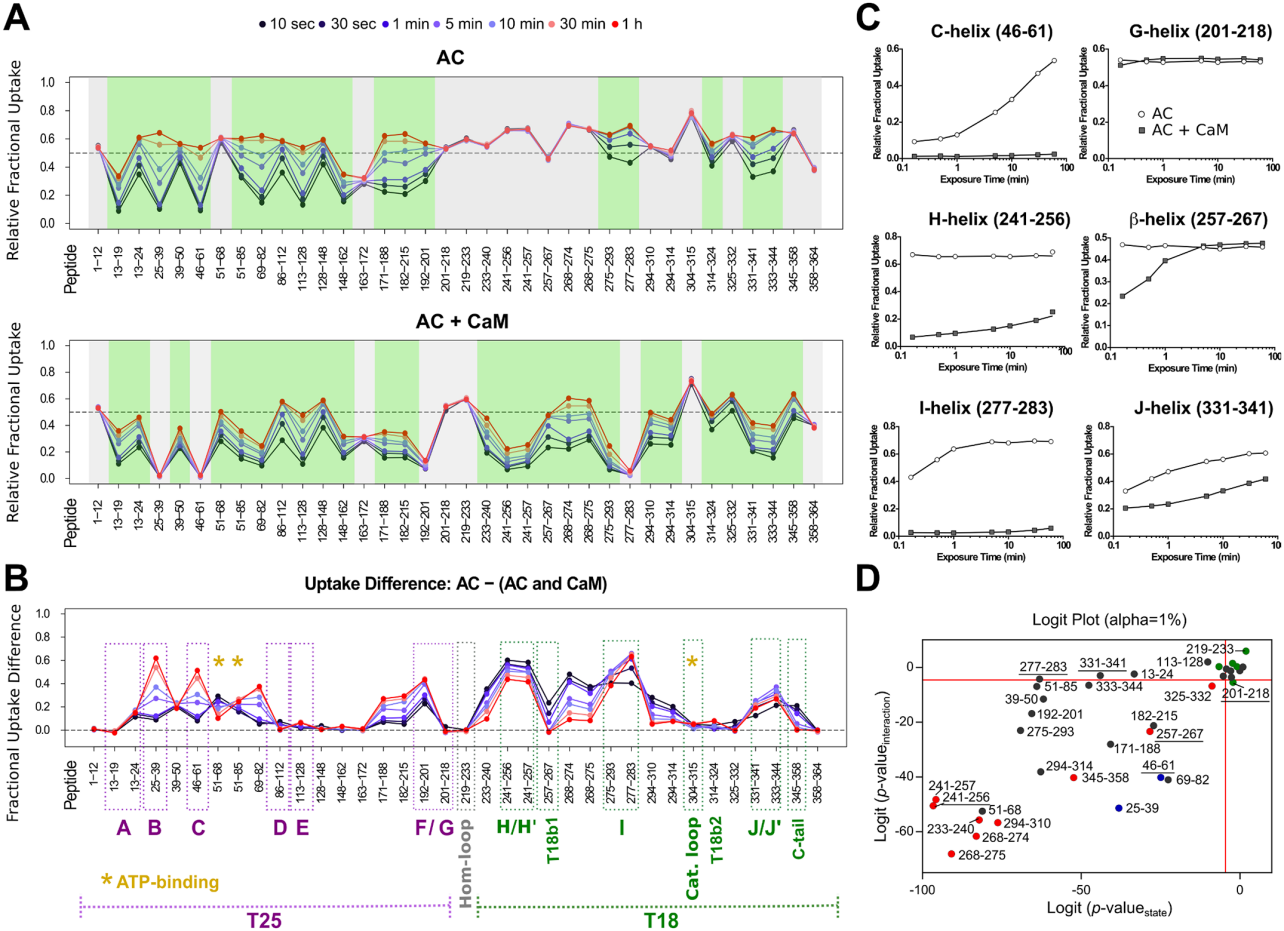
AC dynamics are significantly altered upon CaM interaction. HDX-MS was performed at the peptide level to improve the spatial resolution. Panel (A) displays relative fractional uptake maps of AC alone and upon CaM binding. The presence of dynamic HDX-MS indicative of structure formation is colored green, whereas non-dynamic events are colored gray. CaM binding to AC results in an overall reduction in the solvent accessibility and structure formation in several regions. (B) Multiple regions of AC are altered upon CaM binding, as determined by the uptake difference chart. Major modifications of the AC T25 region are observed within helices B, C, and F. For the T18 region, primary sites of alteration are found within the H/H″-, I-, and J/J′-helices as well as within the T18b1 and C-tail regions of the protein. No changes in accessibility were observed at the Hom- and catalytic loops in either state. Sites of ATP-binding are also given. (C) Several types of HDX-MS behavior were observed upon AC:CaM complex formation. All AC peptides selected for final HDX-MS analysis are displayed in S10 Fig. (D) Logit representation of the statistical results generated for each peptide by MEMHDX. Peptides colored red display nondynamic behavior in the AC alone state only, while those in blue give nondynamic behavior in the AC + CaM state only. Green peptides are those that have nondynamic HDX-MS behavior in both states, while those peptides colored black are dynamic in both states. The FDR value was set to 0.01 (red lines). The data used to generate the figure can be found in S1 Data. AC, adenylate cyclase catalytic domain; CaM, calmodulin; FDR, false discovery rate; MEMHDX, Mixed-Effects Model for HDX experiments; T18, C-terminal trypsin-cleavage fragment of CyaA (amino acids 225–364); T25, N-terminal trypsin-cleavage fragment of CyaA (amino acids 1–224); T18b1, first beta-sheet of the T18 fragment.

We therefore undertook a second cycle of SAXS modeling by incorporating the structural data deduced from HDX-MS and far-ultraviolet (far-UV) SR-CD data. Regarding local-level HDX-MS analysis, pepsin digestion of AC yielded 181 unique peptides identified from their accurate masses and production spectra. A total of 36 peptides covering 100% of the AC sequence were included in the final data analysis (S1 Fig; the nomenclature of secondary structures from W.J. Tang [7] is used throughout this work and reported in the legend of S1 Fig). HDX-MS experiments performed on the isolated AC protein show a significant difference in structural content between the N-terminal (T25 trypsin-cleavage fragment of CyaA, from residues 1–224) and C-terminal (T18 trypsin-cleavage fragment of CyaA, from residues 225–364) regions of the protein (Fig 2A, top panel; S1 Data). Dynamic HDX-MS behavior, indicative of the presence of secondary structural elements, was observed throughout the N-terminal moiety of AC (specifically, in A–E-helices and the beta-sheets of T25), confirming that T25 domain is well folded. Two peptides, namely 51–68 and 163–172, give no dynamic HDX-MS pattern and correspond to loops between structural elements. The C-terminal moiety, on the other hand, appears poorly structured: the few regions exhibiting dynamic HDX-MS behavior cover the I- and J/J′-helices and the second beta-sheet of the T18 fragment (T18b2) region, respectively. Strikingly, the amide hydrogens from the region covering residues 201–275 are fully exchanged from the first time point of the HDX experiment to the last, indicating that this region of circa 75 amino acids is essentially disordered (Fig 2A and 2C). This result provides direct evidence that the F-, G-, H-, and H′-helices, the Hom-loop, and the first beta-sheet of the T18 fragment (T18b1) are disordered in the isolated AC protein. This is somewhat unexpected, as in the X-ray structure of AC:C-CaM, this region is predominantly helical [7]. Finally, the catalytic loop (residues 300–315) is fully exposed to the solvent.

In view of the major differences observed between our HDX-MS results and the crystal structure in a helix-rich part of the crystal structure, we decided to complement our experimental approach using the spectroscopic far-UV SR-CD method, which constitutes a very sensitive probe of a protein helical content in solution. The helical content of AC was estimated from the SR-CD spectrum (S2 Fig) using the BestSel software [12] and compared to that of the crystal structure using the Dictionary of Secondary Structure of Proteins (DSSP) algorithm [13, 14] (S2–S4 Tables and S2 Data). The analysis of SR-CD data indicate that 27% of all residues are part of helical secondary structures, a value significantly lower than the 36% value derived from the X-ray structure using DSSP. However, the SR-CD value favorably compares with HDX-MS results, in which the region corresponding to F-, G-, H- and H′-helices and the T18b1 appear to be disordered in solution (Fig 2A).

Taking into account these results from HDX-MS and far-UV SR-CD, the residues from 200–270 were released and allowed to move in SAXS modeling to obtain a new set of models and associated fits, an example of which is shown in Fig 1B (χ^2^ = 1.36). The distributions of reduced residuals corresponding to the two fits are shown in Fig 1C, while the conformation with relaxed F–H′-helices is shown in red in Fig 1D. This model exhibits a much better fit to the SAXS data and also displays a helical content close to that experimentally determined by SR-CD (S2 Fig and S2 Table). Overall, our model of AC in solution accounts for all three sets of experimental data.

### Structural model of CaM

**Fig 3:**
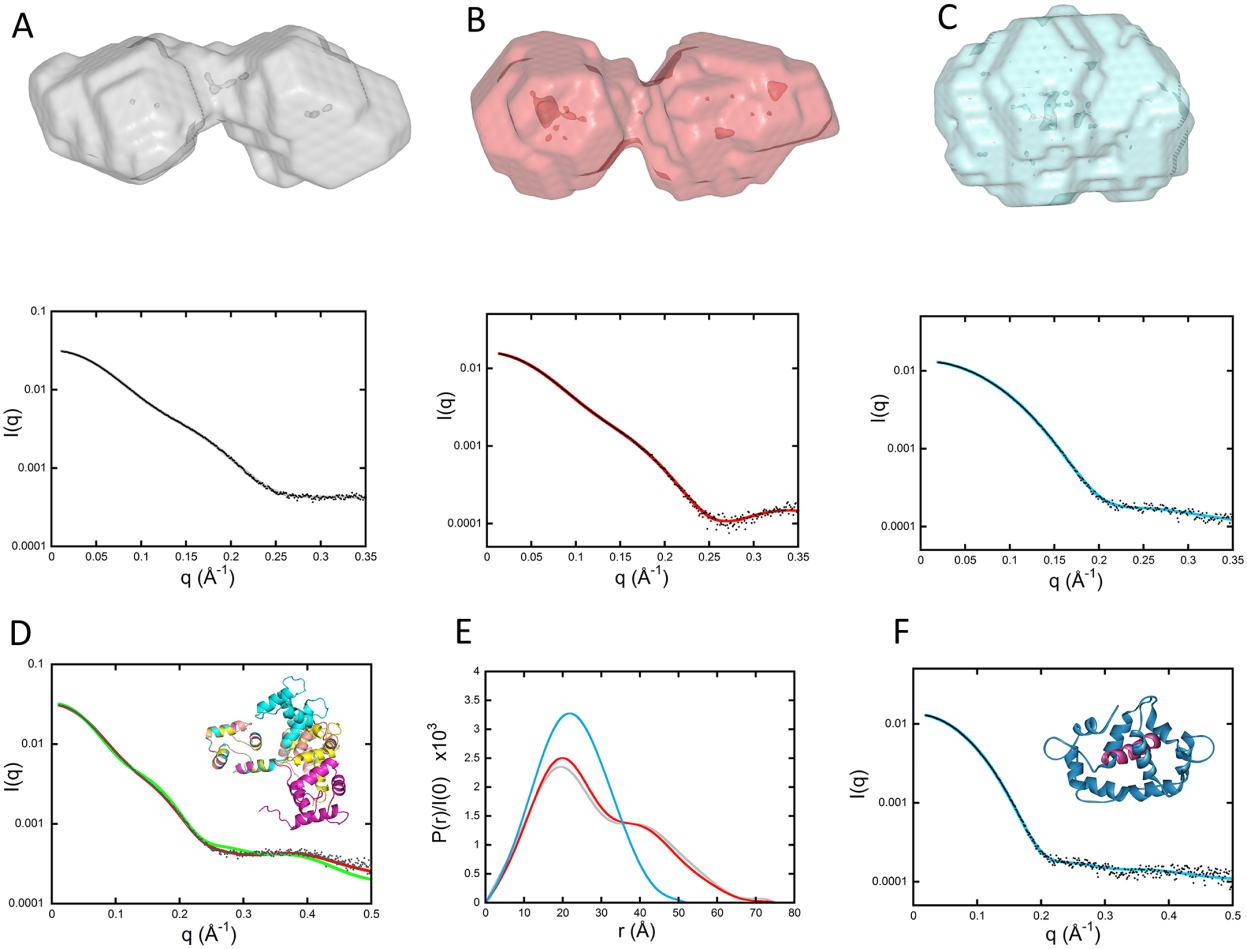
SAXS envelopes of CaM alone and in the presence of H-helix and P_MLCK_ peptides. (A, B, and C [top panels]) DAMMIN models of CaM alone, CaM:H-helix, and CaM:P_MLCK_ complexes, respectively. (A, B, and C [bottom panels]) Corresponding fits (color curves) to experimental data (black dots). (D) Green curve: comparison of experimental data (black dots) to the scattering pattern of the crystal structure of CaM (pdb 1CLL) calculated using Crysol. Red curve: fit obtained using the program EOM and corresponding to the ensemble of four conformations shown in the inset after superimposition of the N-terminal domain of each conformation. (E) Comparison of the three distance distribution functions obtained using the program GNOM for CaM alone (grey), CaM:H-helix (red), and CaM-P_MLCK_ (cyan) complexes. (F) Comparison of experimental data (black dots) to the scattering pattern of the crystal structure of CaM: P_MLCK_ (pdb 2K0F shown in the inset) calculated using Crysol (blue line). The P_MLCK_ peptide is shown in purple. CaM, calmodulin; EOM, Ensemble Optimization Method; pdb, Protein Data Bank; P_MLCK_, myosin light-chain kinase peptide; SAXS, small-angle X-ray scattering.

**Fig 4:**
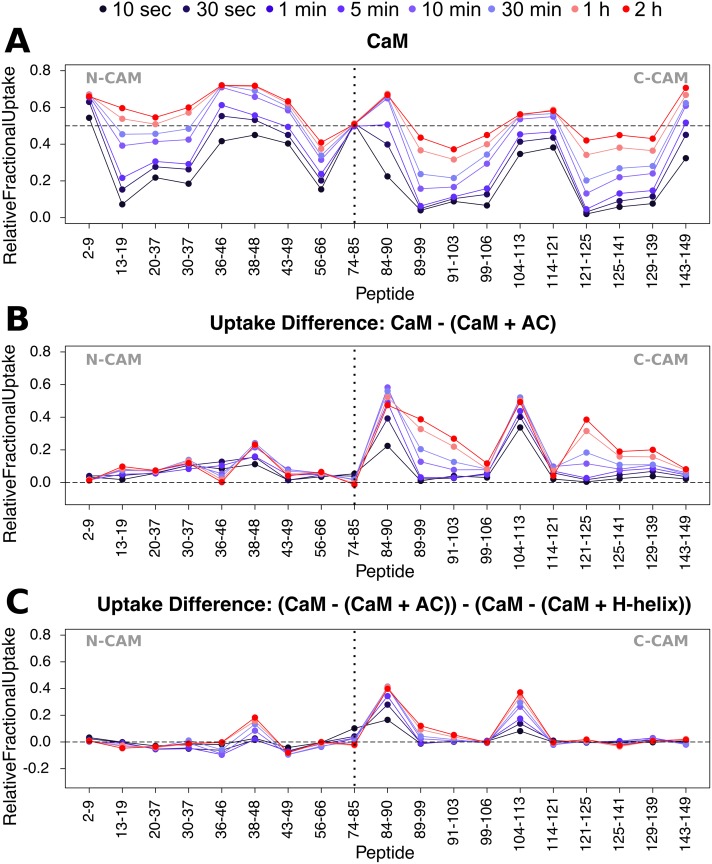
Differential HDX-MS patterns within CaM upon H-helix and AC binding. (A) Relative fractional exchange data were calculated at each point and plotted as a function of peptide position for full-length CaM. (B) Uptake difference plot for CaM in the presence of AC. AC-induced differences in deuterium exchange are principally located in C-CaM. (C) Uptake “difference of uptake differences” plot of CaM in the presence of AC and H-helix peptide. Addition of the full-length catalytic domain results in much greater differences in the deuteration of both N- and C-CaM compared to the H-helix region alone, e.g., in peptides 84–90 and 104–113. Uptake plots of all CaM peptides selected for final HDX-MS analysis are given in S11 Fig. The data used to generate the figure can be found in S3 and S4 Data. AC, adenylate cyclase catalytic domain; C-CaM, C-terminal domain of CaM; CaM, calmodulin; HDX-MS, hydrogen/deuterium exchange mass spectrometry; N-CaM, N-terminal domain of CaM.

CaM has been the object of many SAXS studies over the last few decades [15–19]. Ab initio modeling of our CaM data using the DAMMIF/DAMMIN programs [20] yields the well-known dumbbell shape shown in Fig 3A. Yet, the experimental scattering pattern differed significantly from that calculated from the crystal structure of CaM (1CLL [21]) (Fig 3D, χ^2^ = 37, green curve)—not surprisingly, given the flexibility of the inter-domain helix, as revealed previously by nuclear magnetic resonance (NMR) studies and corroborated by our present HDX-MS data (see Fig 4, described later). This flexibility makes more difficult the ab initio modeling of the protein on the basis of the SAXS data. Hence, CaM was first described as an ensemble of conformations using the Ensemble Optimization Method (EOM) suite of programs [22]. Ten thousand conformations were generated by the Ranch program, starting from the structures of each Ca^2+^-bound domain determined in solution from NMR data by Chou and colleagues (pdb files 1J7O and 1J7P for N- and C-terminal domains, respectively [23]). Residues in the center of the inter-domain helix were described as dummy residues with variable positions. These dummy residues were substituted by a full-atom description using the program PD2 [24]. At this point, the Gajoe program was used to select ensembles of conformations, the average scattering pattern of them being fitted against our experimental data using a genetic algorithm. Fig 3D shows a typical ensemble of conformations that yields a good fit to the experimental data (χ^2^ = 2.15, red curve). This ensemble of conformations illustrates that CaM is made of two folded lobes connected by a highly flexible hinge.

Pepsin digestion of CaM yielded 64 unique peptides, covering approximately 88% of the protein sequence. From these, 20 peptides were selected for HDX-MS analysis (S3 Fig). The dynamic HDX-MS pattern of the isolated CaM protein is typical of a folded protein (Fig 4A, S4 Fig and S3 Data). Notably, however, the central part of the inter-lobe helix (residues 66–92, represented here by peptide 74–85) is fully exchanged, indicating that these residues do not adopt a stable helical conformation but rather behave as a flexible link between the N-terminal domain of CaM (N-CaM) and C-CaM. The α-helical structural content of the isolated CaM protein inferred from SR-CD experiments (S2 Fig, S2 Table, and S2 Data) is 51 ± 3%. This is, however, significantly lower than that observed in the CaM X-ray structure (62%) and most likely due to the disordered segment within the central part of the inter-lobe helix observed in our HDX-MS results.

### CaM:H-helix complex

The H-helix region of AC represents the primary site of interaction with CaM, as seen in the crystal structure of AC with C-CaM [7]. Accordingly, the complex of CaM with the H-helix peptide was investigated in parallel. This allowed us to decipher those additional regions of AC that are involved in AC:CaM complex formation and stabilization. Although the joint presence in solution of both 1:1 and 1:2 CaM:H-helix peptide complexes severely limits modeling attempts, ab initio modeling indicated that the global dumbbell shape of CaM was not altered in the complex, as shown in Fig 3B, which presents a typical envelope obtained with DAMMIF/DAMMIN. This is clearly apparent when examining the two distance distribution functions P(r) that exhibit similar maximal extensions with or without the H-helix peptide bound (Fig 3E). These data suggest that H-helix peptide binding does not cause any significant modification of the CaM dumbbell shape and its inter-domain helix.

Far-UV SR-CD indicates that more than 20 amino acids undergo a disorder-to-helix transition upon CaM:H-helix complex formation (S2 Fig, S4 Table and S2 Data). The binding of the H-helix peptide does not create any additional dynamic HDX-MS activity within CaM (S4 Fig and S4 Data). Consequently, the observed increase in helical content can be attributed to the disorder-to-order transition within the H-helix peptide itself. The H-helix peptide appears to stabilize the C-CaM helices of CaM (Fig 4C and S4 Fig) and to acquire a helical conformation upon CaM binding (S2B Fig, S2 and S4 Tables).

### CaM:P_MLCK_ complex

Ab initio modeling of SAXS data indicated that the global dumbbell shape of CaM was not altered upon binding of the H-helix peptide. We therefore undertook equivalent experiments on the CaM protein in complex with the myosin light-chain kinase peptide (P_MLCK_), which is known to reshape CaM from its extended dumbbell conformation into a compact globular structure. In marked contrast to the H-helix peptide, the shape of the CaM:P_MLCK_ complex determined ab initio using the programs DAMMIF/DAMMIN was clearly globular and isometric (Fig 3C). Noticeably, the experimental scattering curve of CaM:P_MLCK_ is very similar to the scattering pattern calculated from the known crystal structure of this complex (2K0F.pdb) (Fig 3F), in which the two domains of CaM are wrapped around the P_MLCK_ peptide. Altogether, these SAXS studies confirm the distinct modes of interaction of CaM with the H-helix peptide of AC and the P_MLCK_ peptide from MLCK. This difference is also clearly evident when comparing the distance distribution functions P(r) (Fig 3E): CaM:P_MLCK_ exhibits a bell-shaped curve with a single maximum, while the CaM:H-helix profile presents an extra shoulder characteristic of a two-domain structure.

Finally, P_MLCK_ binding exerts a more pronounced reduction in solvent accessibility than the H-helix throughout the full-length CaM protein, with both N- and C-CaM lobes affected (S5 Fig and S5 Data). However, the linker connecting both domains remains solvent exposed throughout, as previously observed with both the H-helix peptide and the full-length AC domain.

### AC-CaM complex in solution

The HDX-MS data indicate that the binding of CaM induces several statistically significant (*p* ≤ 0.01) changes in AC (Fig 2A and 2D and S1 Data). Globally, the N-terminal moiety is stabilized, while the C-terminal domain now exhibits several regions characterized by HDX dynamic patterns, indicative of secondary structure acquisition within T18. The main effects in the N-terminal part of AC are the stabilization of both B- and C-helices and of the embedded T25 antiparallel beta-sheet (peptides 25–39, 46–61, and 192–201), as highlighted in Fig 2B. These structural elements become completely resistant to deuteration over the experimental timescale (Fig 2A and 2C). The internal loop (51–68), which was fully accessible in the isolated AC, exhibits a dynamic HDX-MS behavior in AC:CaM. In contrast, the regions corresponding to the G-helix as well as the Hom-loop are unaffected by CaM binding, suggesting that these solvent-exposed regions in the isolated AC protein remain accessible throughout (Fig 2A–2C). Upon complex formation, the majority of the C-terminal half of AC acquires secondary structure elements, except the catalytic loop (residues 304–315 from CR3), which remains completely solvent exposed. Most notably, the regions encompassing the H/H′-helices and T18b1, which were fully exposed in AC alone, are structured in the presence of CaM (see dynamic HDX behavior in Fig 2A–2C). The I- and J/J′-helices, which were already structured in the absence of CaM, are further stabilized, as evidenced by the observed reduction in deuteration level.

Reciprocally, we examined the consequences of AC binding for CaM structural dynamics. HDX-MS data show that AC binding induces a strong stabilization of C-CaM, as evidenced by the dramatic reduction in accessibility imposed by the wrapping of C-CaM helices around the H-helix region of AC (Fig 4B and S6 Fig). The loop between the first two helices, H5 and H6, of C-CaM is also significantly stabilized, likely via interactions with the J/J′-helices and the C-terminal loop of AC. The involvement, beyond the CBS-containing H-helix, of various regions of AC in CaM stabilization is best illustrated by the “difference of uptake differences” plot (Fig 4C). In contrast, the majority of N-CaM is unaffected, besides peptide 38–48, which covers the loop connecting helices H2 and H3. Notably, the central part of the inter-lobe helix of CaM is also unaffected by AC binding and remains solvent exposed upon complex formation.

Finally, we recorded the far-UV SR-CD spectrum of the AC-CaM complex (S2 Fig) to estimate the CaM-induced helical content increase of AC. Assuming a constant helical content of CaM, we derive for AC an increase in its helical content from 27% to 42 ± 3% upon CaM binding (S2–S4 Tables). This increase, corresponding to about 40 amino acids (S3 Table), is proposed to be primarily due to the CaM-induced folding of the H/H′ regions, as reported by the HDX-MS results (Fig 2).

The first modeling attempt of the AC-CaM complex from our SEC-SAXS data made use of the BUNCH program, which considers that the particle adopts a unique conformation in solution and searches for one conformation that yields a good fit to the experimental data. More specifically, AC and each CaM domain were handled as rigid bodies while the program searched an optimal conformation of the inter-domain helix of CaM, the only variable part of the complex. We started from the crystal structure 1YRU of the complex of AC with the C-CaM and the solution structure 1J7O of the N-CaM. Furthermore, the central residues of the inter-domain helix were described as dummy residues, the positions of which were varied by the program in order to adjust the calculated scattering pattern to experimental data. We made one change to the 1YRU structure based on our experimental HDX-MS data: residues 200–234, corresponding to helices F and G together with the disordered Hom-loop, remain unfolded in the AC:CaM complex. Indeed, examination of the crystal structure and of its contacts with neighboring molecules shows that these two helices are interacting with their counterpart from an adjacent molecule within the crystal. Moreover, these residues are not predicted to adopt a helical conformation by secondary structure predictions. Accordingly, we assumed that the two helices are strongly stabilized within the crystal but may only transiently exist in solution. Therefore, we released the position of residues 200–234 of AC so that this stretch was also variable during the refinement process. Thirty runs of the program BUNCH yielded models similar to that shown in S7 Fig. The corresponding fit to the experimental data is also shown in S7 Fig (χ^2^ = 2.0). Noticeably, the N-CaM is immersed in the solvent at a distance from the rest of the complex, without any contact with AC (S7 Fig).

**Fig 5:**
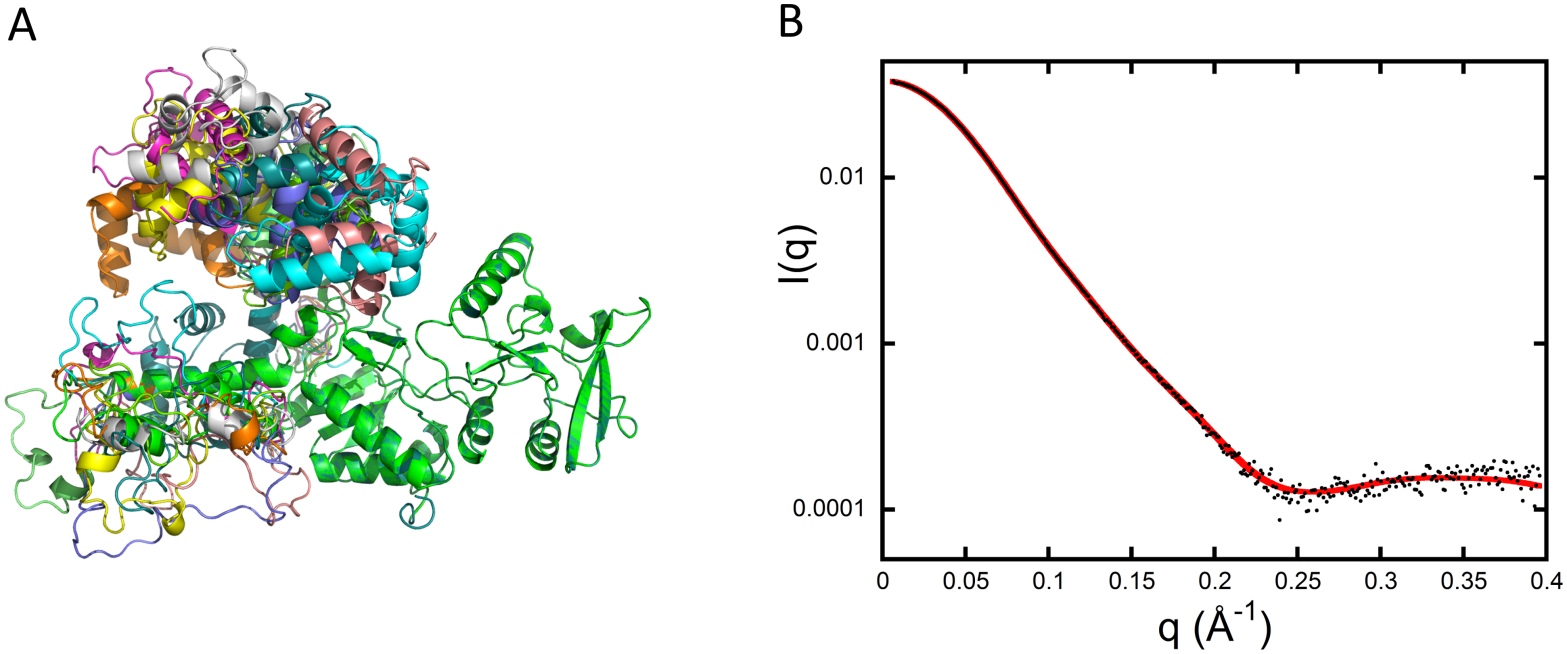
Modeling of the AC:CaM complex. (A) Typical ensemble of conformations describing the AC:CaM complex, obtained using the program EOM and displayed after superimposition of the AC moiety of each conformation (green) (see main text for details). (B) Corresponding fit (red curve) to experimental data (black dots). AC, adenylate cyclase catalytic domain; CaM, calmodulin; EOM, Ensemble Optimization Method.

The high flexibility of the central CaM linker suggests that a better description of the complex would make use of an ensemble of conformations (as opposed to a unique structural model). Accordingly, we undertook a second round of complex modeling using the program EOM. The 10,000 conformations of the initial pool differ in the relative position of the two CaM domains as well as in the conformation of the region 200–234 of AC. An example of the resulting ensemble of conformations is displayed in Fig 5A, with the corresponding fit to the SAXS data shown in Fig 5B (χ^2^ = 1.41). Interestingly, the SAXS data are compatible with a description of the complex in which the flexibility of the CaM inter-domain helix makes possible transient interactions of the CaM N-terminal domain with AC, as suggested by the results of HDX-MS experiments (CaM peptide 38–48, see Fig 4B).

## Discussion

**Fig 6:**
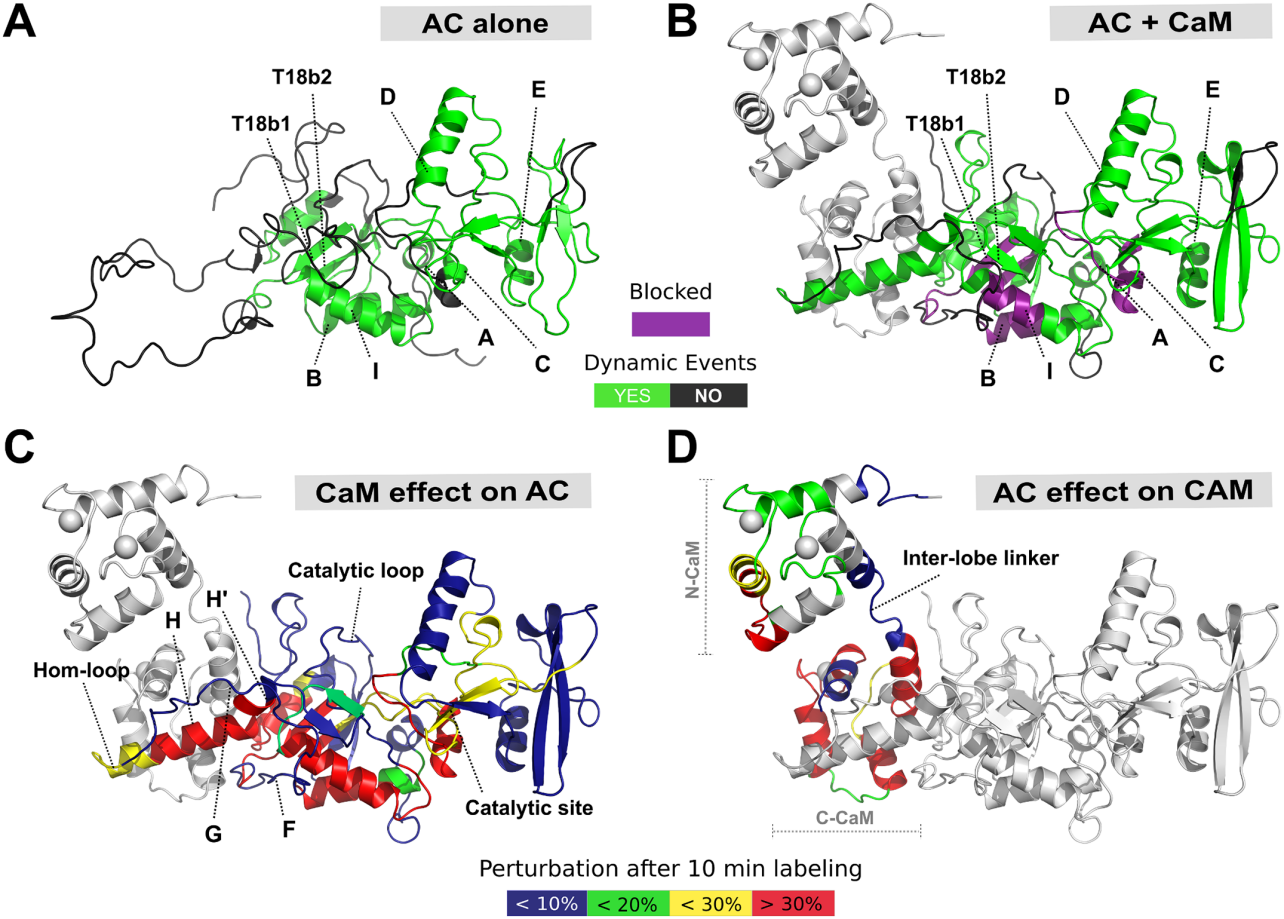
The structural interplay of AC and CaM complex formation. Specific regions within the AC T18 domain serve as a MoRF for CaM recognition, binding, and activation of AC itself. (A) In the absence of CaM, the F-, G-, H-, and H′-helices and Hom-loop are found as an extended disordered coil, acting as a bait for CaM capture. (B) An interplay between protein structural disorder and order is requisite for activation by CaM of AC catalytic function. Upon CaM binding, the H- and H′-helices undergo extensive structure formation, resulting in a conformation that is appropriate for catalytic activation. Helices F and G and the Hom-loop remain unstructured throughout. Some regions become “blocked” and resistant to deuteration (highlighted in purple). (C) The effect of CaM binding on AC. (D) The effect of AC on CaM. CaM binding to AC results in widespread perturbations, primarily within the T18 region of the protein, while AC primarily binds to C-CaM, with only a transient interaction in N-CaM. In addition to those effects occurring in the H/H′ region, long-range allosteric remodeling is observed at the site of catalysis, which becomes more stable and rigid. Meanwhile, the catalytic loop does not undergo any dramatic structural rearrangement, remaining unstructured and exposed regardless of CaM availability. This is suited to a maximal turnover of ATP substrate and thus maximal toxicity in the form of cAMP production. The data used to generate the figure can be found in S1, S3 and S4 Data. AC, adenylate cyclase catalytic domain; C-CaM, C-terminal domain of CaM; CaM, calmodulin; MoRF, molecular recognition feature; N-CaM, N-terminal domain of CaM; T18, C-terminal trypsin-cleavage fragment of CyaA (amino acids 225–364); T18b1, first beta-sheet of the T18 fragment; T18b2, second beta-sheet of the T18 fragment.

Exploiting an integrative structural biology approach, the present study proposes detailed and novel structural descriptions of the catalytic AC domain of the CyaA toxin, both in isolation and in the presence of its activator, CaM, and describes how specific conformational rearrangements within each molecule may be requisite to trigger the enzymatic activity. The isolated AC protein appears significantly less folded in solution than in the crystal structure of the AC:C-CaM complex [7]. Although the N-terminal T25 domain is folded, the C-terminal T18 moiety contains few structural elements: most noticeably, HDX-MS identifies a large region of 75 amino acids (residues 201–275) in T18, which is predominantly structurally disordered. This IDR includes the H-helix region, which is the main CBS of AC, as well as its flanking regions, namely the Hom-loop and the F-, G-, and H′-helices (Fig 6A). This is in agreement with prior studies that showed that the isolated AC domain is partially folded [25–27].

AC:CaM complex formation primarily takes place between the H/H′-helix region of T18 and C-CaM, inducing the folding of T18 as well as establishing long-distance stabilization of structural elements within the T25 moiety (Fig 6B and 6C, S8 and S9 Figs; [7, 28–30]). The SR-CD and HDX-MS data of both AC:CaM and H-helix:CaM complexes strongly suggest that CaM binding triggers the folding of the H- and H′-helices (S1–S4 Tables). However, the area upstream of the H-helix region clearly remains highly flexible in the AC:CaM complex. Several regions of T18, including the I- and J/J′-helices (residues 273–290 and 331–344, respectively) as well as the C-terminal LHL motif (residues 345–358, which interact with the AC catalytic loop [8]) are significantly stabilized upon CaM binding. These different interactions may contribute to the shaping of the catalytic site through the correct positioning of the CR3 loop, which directly interacts with the purine moiety of the ATP substrate. CaM binding also induces significant modifications in the T25 region of AC, including the B- and C-helices, and the conserved catalytic regions CR1 and CR2, which contact the ribose and triphosphate moieties of ATP [7] (Figs 2 and 6). In contrast, the G-helix region remains highly flexible in the AC:CaM complex in solution, and it is likely that the helix is stabilized in the crystal structure of AC:CaM through interactions with its counterpart in symmetry mates. Our experimental SAXS data are indeed fully compatible with AC:CaM models in which all AC residues from 200–234 remain largely disordered.

The significant level of structural disorder observed in AC alone, with a large IDR of about 75 residues, has major implications for the biological functions of the molecule. In the first instance, the IDR and global structural disorder of the T18 moiety may contribute via an “entropic chain effect” to destabilizing the catalytic site and thus preventing cAMP synthesis in the absence of the activator—most importantly, at the time of CyaA production in *B*. *pertussis*, when any significant basal activity would be deleterious to the bacterial host, as illustrated by the high toxicity observed when CaM and AC are co-expressed in recombinant *Escherichia coli* [31]. Secondly, the weak stability of significant parts of the AC structure is favorable for toxin delivery from the bacterial host to eukaryotic target cells. CyaA is secreted outside the bacteria by a type I secretion system (T1SS), and then, upon binding to target cells, AC is delivered across the plasma membrane to reach the eukaryotic cell cytosol [32]. There is evidence to suggest that both the secretion through the T1SS and the translocation across the eukaryotic cell membrane are critically dependent upon the ability of the polypeptide chain to adopt partially unfolded states [5, 33–36]. The disordered region and overall low structural content of AC in solution should clearly facilitate these key steps of the intoxication process. Thirdly, the intrinsic disorder of the central region of AC, which harbors the main CBS, namely the H-helix, potentially plays a crucial role in molecular recognition of the activator [37, 38]. Indeed, the CBS located within the IDR of AC (see the long loop in Fig 6A) exemplifies the canonical properties of so-called molecular recognition features (MoRFs) or short linear motifs (SLIMs), which are short and conserved motifs involved in recognition (in silico analysis in S8 Fig). MoRFs often comprise a few hydrophobic residues amidst charged residues and are frequently embedded within IDRs that serve as flexible linkers to efficiently expose the MoRFs to the solvent, thereby favoring their potential interactions with partner biomolecules [37]. The MoRF in AC is typical of eukaryotic CaM-binding targets: it is 10–20 residues long and is flanked by disordered regions, with a high net charge and a propensity for structural disorder in the CaM-free state (S8 Fig). Moreover, it undergoes a disorder-to-helix transition upon complex formation [39]. Such coupled binding and folding processes appear to be a common mechanism by which CaM activates its target enzymes, including, for example, the CaM-dependent protein kinases and calcineurin [40]. The AID of these latter proteins occludes access to the catalytic site in the resting state. A CaM-induced conformational change physically shifts the AID from the enzymatic site, allowing catalysis. In the case of AC, the structural disorder of the catalytic site induced by the IDR replaces the steric hindrance of the AID.

Once AC has been delivered across the target cell plasma membrane, the long IDR may facilitate the efficient capture of the intracellular CaM through a “fly-casting” mechanism [9]. CaM binding promotes folding of the H/H′ region and stabilization of several structural elements of T18 (Figs 2 and 6). The reduction of disorder is then propagated to the core of the T25 domain to allosterically stabilize other key regions shaping the catalytic site (Fig 6B and 6C). This suggests that the overall dampening of disorder triggered by CaM-binding is crucial for AC activation. Interestingly, our HDX-MS data indicate that, although CaM binding imposes a strong entropy reduction and steric restrictions on regions flanking the CR3 catalytic loop, the loop itself (residues 300–314) remains largely dynamic and solvent exposed, as indicated by the rapid and high deuterium uptake (S10 Fig). The high flexibility and structural dynamics of this catalytic loop that binds the purine ring of ATP are likely crucial for the efficient catalysis of AC, which has a turnover number higher than 1,000 s^-1^, i.e., one ATP converted to cAMP in less than a ms.

A striking property of AC is that it is fully activated by the C-terminal lobe of CaM, with an affinity that is only 10–20-fold lower than that of the full-length CaM protein [7]. As only the structure of AC in complex with C-CaM has been reported thus far, the structure and mode of interaction of AC with the full-length CaM molecule has remained elusive. Our HDX-MS analysis indicates that the C-CaM lobe is strongly stabilized upon complex formation (Fig 6D and S11 Fig), while the N-CaM domain remains mostly dynamic and essentially unaffected by the presence of AC (Figs 4 and 5). This is in good agreement with the Springer and colleagues NMR study, which also showed that AC binding primarily affects C-CaM, with minimal structural changes to N-CaM [41]. Notably, when bound to either AC or the H-helix peptide, CaM remains in an extended conformation (Figs 3 and 5), with high flexibility of the central CaM inter-lobe helix (Fig 4, S4 and S6 Figs). Our SAXS data are in excellent agreement with structural models in which CaM interacts with AC essentially via its C-terminal lobe, while its N-terminal lobe, connected through the flexible central linker region (residues 77–83), remains highly dynamic, without establishing stable contacts with AC (Fig 5), although transient interactions are likely, as suggested by both our EOM analysis of SAXS data and our HDX-MS results. Interestingly, these models are reminiscent of those proposed by Guo and colleagues [30] on the basis of molecular dynamics simulations, although these authors further suggested that the β-hairpin region (residues 259–273) of AC directly contacts the second calcium-binding motif of the extended CaM. To probe this model, the group performed cross-linking experiments between AC and CaM variants harboring a unique cysteine at positions 260 and 50, respectively (AC-Q260C and CaM-D50C). However, only a very faint disulfide bond cross-link was detected, indicating that the proposed conformations with the two cysteines in proximity are weakly populated in solution. From our point of view, these data support the view of a fairly dynamic N-CaM lobe that samples a variety of conformations, restricted only by the flexibility of the central hinge (Fig 4). How the N-CaM lobe may contribute to the 10–20-fold higher affinity of native CaM as compared to that of C-CaM [42, 43] remains an intriguing question. One attractive hypothesis could be that the mere presence of the N-CaM module, acting as a “local crowder,” may stabilize the complex by restricting the conformational dynamic of the AC polypeptide chain (mainly the T18 region). Additional stabilization may also be provided by global electrostatic interactions between the overall negatively charged N-CaM and the positively charged cluster of residues around 221–224 of AC [29].

Interestingly, our combined SAXS, HDX-MS, and SR-CD analyses also show that the CaM dumbbell shape [44, 45] is not significantly modified upon binding to the AC H-helix peptide. This is in marked contrast to what is observed with the classical CaM-binding peptide from MLCK, P_MLCK_ (Fig 3), around which both N- and C-CaM are seen to wrap in a conformation that requires a sharp bending of the CaM central helix (Fig 3C, S5 and S12 Figs) [46]. The ability of individual N- and C-terminal lobes of CaM to independently associate to either distinct sites on the same protein or to the same site on two distinct polypeptides, thus facilitating their dimerization, have been described for numerous CaM targets, although in many instances, the affinity of individual lobes with their target sequence is lower than that with the AC H-helix. Our data further highlight the remarkable conformational diversity of CaM upon binding to its targets [44–49].

## Conclusions

In the bacterial cytosol, there is no chaperone, inhibitor, or antitoxin available to prevent cAMP production, and thus we propose that structural disorder acts as the inhibitor of CyaA catalytic activity. Once translocated into eukaryotic cells, bacterial toxins such as CyaA hijack CaM, which is used as a disorder dampener to activate the enzyme. The disorder-to-order transition observed in the MoRF region of AC is not directly involved in catalysis but rather transduces the stabilization toward the enzymatic region. This CaM-induced folding and stabilization, from local to distal regions, allosterically tunes the enzymatic activity of bacterial toxins.

## Materials and methods

Buffer A composition is 20 mM HEPES, 150 mM NaCl, pH 7.4, and 2 mM (HDX-MS and SR-CD experiments) or 4 mM (SEC-SAXS experiments) CaCl_2_ (final calcium concentration), unless otherwise stated.

### Protein production and purification

The H-helix peptide corresponds to residue numbers 234–254 of AC (LDRERIDLLWKIARAGARSAVG). H-helix peptide molecular mass deduced from mass spectrometry (MS) was 2,509 g/mol, its pI was 11 and it contained +2 charges at neutral pH, and its molar epsilon at 280 nm was 5,600 M^−1^ cm^−1^. The CaM-binding peptide (P_MLCK_) from the smooth muscle MLCK corresponds to residues RRKWQKTGHAVRAIGRLSS (Em: 5,600 M^−1^ cm^−1^). All peptides contain an N-terminal acetyl and C-terminal amide as capping groups. All peptides were purchased from Genosphere Biotechnologies (Paris, France).

Human CaM protein was produced in *E*. *coli*, as previously described [27]. MS confirmed the integrity and identity of all proteins in the study. MS analysis of CaM gave a single peak of 16,706.25 Da (expected mass = 16,706.30 Da). A molar epsilon at 280 nm of 2,980 M^−1^ cm^−1^ and a pI of 4.1 were derived from the CaM sequence using ProtParam.

The adenylate cyclase catalytic domain (AC) toxin under investigation corresponds to residues 1−364 of *B*. *pertussis* CyaA. The protein was produced in *E*. *coli* and purified as follows: protein-containing inclusion bodies from the bacterial cell pellet were resuspended in 6 M GdnHCl, 20 mM HEPES, pH 7.4, overnight at 4°C, and then buffer exchanged from 6 M GdnHCl to 6 M urea on prepacked G25SF desalting columns. Inclusion body solubilization was performed in GdnHCl instead of urea to prevent AC364 carbamylation. AC364 was purified by two successive chromatographic separations on DEAE-Sepharose [4]. The protein was initially eluted in 8 M urea, 20 mM HEPES, and 500 mM NaCl, pH 7.4, followed by a second elution in 20 mM HEPES and 100 mM NaCl, pH 7.4. AC364 was further purified on a Sephacryl S200 column equilibrated with Buffer A and concentrated on an Amicon 10 kDa MWCO filter. The identity and purity of protein batches was monitored by SDS-PAGE and MS. AC364 gave a single MS peak of 39,380.90 Da (expected mass = 39,381.50 Da). A molar epsilon at 280 nm of 28,880 M^−1^ cm^−1^ and a pI of 6.2 were derived from the AC364 sequence. All proteins and peptides, whether in solution or lyophilized, were stored at −20°C.

### HDX-MS

#### Sample preparation

Five experimental conditions were analyzed. In the first instance, AC was incubated in both the presence and absence of a twofold molar excess of CaM. Furthermore, CaM was incubated alone or in a twofold molar excess of either H-helix or P_MLCK_ peptide. The excess of peptide or protein ensured that all complexes were fully formed and maintained over the labeling reaction (AC:CaM K_D_ < 0.1 nM, P_MLCK_:CaM and H-helix:CaM K_D_ are in the nM range). Prior to addition of the deuterated buffer, all solutions were equilibrated for 1 h at room temperature. Continuous labeling was performed at 20°C for *t* = 0.16, 0.5, 1, 5, 10, 30, 60, 120, and 240 min in D_2_O containing Buffer A. A D_2_O/H_2_O ratio of 80:20% was selected such that the kinetics favored unidirectional exchange. Aliquots of 10–20 pmol of protein were withdrawn at each time point and quenched upon mixing of the deuterated sample with ice-cold 0.5% formic acid solution to achieve a final pH of 2.5. Quenched samples were immediately snap frozen in liquid nitrogen and stored at −80°C for approximately 12 h. Undeuterated controls were treated using an identical procedure. All MS-analyses were performed in triplicate for each time point and condition.

#### HDX-MS data acquisition

Prior to MS analysis, samples were rapidly thawed. To minimize back exchange, the LC solvent line, injection valve, columns, and sample loop were maintained at 0°C. To achieve this, HDX-MS analyses were performed with the aid of a cooled HDX Manager (Waters Corporation, Milford, MA). Labeled samples were digested using an in-house prepared cartridge of immobilized pepsin beads (Thermo Scientific, Rockford, IL) for 2 min at 70 μL/min and 20°C. Peptic peptides were rapidly desalted, as described above, using a VanGuard C18 pre-column (1.7 μm, 2.1 × 5 mm; Waters) and separated using an ACQUITY UPLC BEH C18 column (1.7 μm, 1 × 100 mm). AC and CaM peptides were separated over an 8-min gradient of 5%–40% acetonitrile (ACN) at 40 μL/min and at 0°C. After each run, the pepsin column was manually cleaned with two washes of 0.8% formic acid, 5% ACN, 1.5 M guanidinium chloride, pH 2.5. Blank injections were performed after each sample to confirm the absence of carryover. The LC flow was directed to a Synapt G2-Si HDMS mass spectrometer (Waters), which was equipped with ESI and lock-mass correction using Glu-Fibrinogen peptide (100 fmol/μL in 50% ACN). Mass spectra were acquired in positive-ion and resolution mode over the m/z range of 50–1,800 using a data-independent acquisition scheme (MS^E^), whereby exact mass information is collected at both low and high collisional energies for collisional-induced dissociation. For local-level analysis, unlabeled AC and CaM samples were digested in triplicate to obtain a peptide coverage map of both proteins.

#### HDX-MS data processing

Peptide identification was via the Protein Lynx Global Server (PLGS) 3.0 (Waters Corporation). Oxidation of methionines and carbamylation of N-terminal and lysine residues were set as variable modifications. The sequence coverage map was plotted using DynamX 3.0 HDX software (Waters Corporation). D_2_O uptake at the peptide level was extracted and visualized in DynamX; only one charge state was considered per peptide. No adjustment was made for back exchange, and the results were reported as relative deuterium exchange levels expressed in either mass unit or fractional exchange. Fractional exchange data were calculated by dividing the experimentally measured uptake by the theoretically maximum number of exchangeable backbone amide hydrogens that could be replaced within each peptide. This number corresponds to the number of amino acid residues present in the peptide minus the number of proline residues and minus one for the N-terminus that back exchanges too rapidly to be measured by MS [50]. HDX-MS results were further analyzed using mixed-effects model for HDX experiments (MEMHDX), using a Wald test (*p* < 0.01) and with a false discovery rate (FDR) of 1% [51].

### Small angle X-ray scattering

#### Data acquisition

X-ray scattering data were collected at the SWING beamline of the SOLEIL Synchrotron (Saint-Aubin, France). All measurements were performed using a size-exclusion HPLC column (Agilent BioSEC-3, 300 Å, 4.6*300 mm) online with the SAXS measuring cell, a 1.5-mm diameter quartz capillary contained in an evacuated vessel [52]. The sample to detector distance was 1,988 mm, allowing useful data collection in the q range 0.006 Å^-1^ < q < 0.5 Å^-1^, for which q = 4πsinθ/λ, 2θ is the scattering angle, and λ = 1.00 Å, the wavelength of the X-rays. We first studied solutions of isolated AC364 and CaM proteins. We then examined solutions of CaM in complex with either H-helix peptide (both 5:1 and 3:1 ratios of peptide:CaM), P_MLCK_ (3:1 ratio of peptide:CaM), or AC364 (1:2 and 1:4 ratios of AC364:CaM). All proteins were analyzed in Buffer A. Scattering of the elution buffer before void volume was recorded and used as buffer scattering for subtraction from all protein patterns. Successive frames of 1 s were recorded and separated by a 0.5-s interval. The elution flow of 0.200 mL/min ensured that no protein was irradiated for more than 0.4 s. For each frame, the protein concentration (about 1–2 mg/mL at the top of elution peak) was estimated from UV absorption at 280 and 295 nm using a spectrometer located immediately upstream of the SAXS measuring cell.

#### Data analysis

Scattered intensities were put on an absolute scale using water scattering. SAXS data were normalized to the intensity of the incident beam and background subtracted using the programs FoxTrot (courtesy of SWING beamline) and Primus (https://www.embl-hamburg.de/biosaxs/primus.html) [53]. Identical frames under the main elution peak were selected using Cormap [54] and averaged for further analysis. The R_g_ was evaluated using the Guinier approximation [55] and also derived from the distance distribution function P(r) calculated by the program GNOM [56]. Two independent determinations of the molecular mass were obtained using the programs SAXSMow2 and ScÅtter, available at URLs http://saxs.ifsc.usp.br/ and https://bl1231.als.lbl.gov/scatter/, respectively. A third determination was also obtained from the value of I(0)/c, in which c is the protein concentration for the two samples containing AC364. This could not be generated for the CaM, CaM:H-helix, and CaM-P_MLCK_ samples, as CaM does not possess any tryptophan. The molecular mass of the CaM/H-peptide complex derived from the SAXS data was intermediate between that of the 1:1 and that of the 1:2 complex. This was confirmed by the estimate of the stoichiometry derived from the amount of free H-helix peptide eluting from the column using the 280-nm elution profiles of the CaM:H-helix complex, CaM alone, and the H-helix peptide alone. A 1:1 stoichiometry was observed for the CaM:P_MLCK_ complex, as judged from the SAXS-derived molecular mass.

### Modeling

#### Ab initio shape determination

Regarding CaM and its complexes with H-helix and P_MLCK_ peptides, the structural effect of peptide binding was investigated by determining the shape or envelope of each scattering particle. For all three systems, 20 shapes were determined using the ab initio program DAMMIF [20] and compared using the DAMAVER suite of routines based on the calculation of the normalized spatial discrepancy (NSD), which is lower than 0.7 if all 20 models are similar. The final envelope is obtained using DAMMIN [57], which uses the envelope of all superimposed DAMMIF models (the union of all models) as initial volume.

#### Rigid-body modeling

All scattering patterns calculated from atomic coordinates were obtained using CRYSOL [58]. AC alone was first modeled using its crystal structure in complex with C-CaM [7]. The scattering pattern was calculated using AllosModFoxs [11], which refines the position of missing residues. For the sake of consistency, the scattering intensities of the resulting models were recalculated using CRYSOL. The complex of AC with CaM was modeled using the BUNCH program [59]. Starting from the crystal structure of AC:C-CaM (1YRU), the program refines the position of the N-CaM domain by modifying the position of the central linker residues so as to fit the data. Modifications to 1YRU were authorized in the 200–234 stretch of AC (helices F and G), a region that appears to be essentially disordered in HDX-MS studies.

#### Ensemble of conformations

Finally, CaM and AC:CaM were modeled in terms of an ensemble of conformations using the program EOM [22]. The routine Ranch first creates a large pool of 10,000 conformations by varying the conformation of the central linker of CaM (hence, the position of the N-CaM domain) and that of the stretch 200–234 of AC (in the case of the complex). The subsequent Gajoe routine determines ensembles of conformations from the pool, the average scattering pattern of which fits our experimental data.

### SR-CD

SR-CD experiments were carried out at the synchrotron facility SOLEIL (DISCO beamline, Saint-Aubin, France). SR-CD spectra were recorded in the far-UV region from 180–260 nm with an integration time of 1.2 s, a bandwidth of 1 nm with a 1 nm resolution-step at 20°C in QS cells (Hellma), with a path length of 50 μm. Each far-UV spectrum represents the average of 3 individual scans. Experimental details on the procedure are described elsewhere [33, 35]. Protein and peptide concentrations ranged from 50–200 μM. The far-UV CD spectra were processed using BestSel to estimate the alpha-helical content of proteins, peptides, and complexes [12].

## Supporting information

S1 Fig Peptide map of AC. A sequence coverage map of AC was determined after 2 min digestion with pepsin. Each blue bar represents a single AC peptide. Only those peptides selected for HDX-MS data analysis after filtering the complete dataset are displayed. Linear sequence coverage of 100% was achieved. The secondary structure architecture of AC is as follows: A-helix (residues 11–17), B-helix (residues 20–34), C-helix (residues 44–53), D-helix (residues 89–106), E-helix (residues 116–126), F-helix (198–210), G-helix (214–223), Hom-loop (residues 226–232), H-helix (residues 234–253), H′-helix (residues 255–259), T18b1 (residues 262–270), I-helix (residues 274–289), catalyic loop (residues 300–312), T18b2 (residues 313–325), J-helix (residues 326–340), and an LHL motif (residues 341–364). The catalytic site is made of three highly conserved regions that are directly involved in substrate binding and catalysis: CR1 (residues 54–77), CR2 (residues 184–198), and CR3 (residues 295–315). AC, catalytic domain; CR1, catalytic region 1; CR2, catalytic region 2; CR3, catalytic region 3; LHL, loop-helix-loop; T18b1, first beta-sheet of the T18 fragment; T18b2, second beta-sheet of the T18 fragment.
(PDF)

S2 Fig Far-UV SR-CD spectra of isolated AC, CaM, H-helix peptide, and their complexes. (A) AC (blue), CaM (red), and AC:CaM complex (dark). (B) H-helix peptide (blue), CaM (red), and H-helix:CaM complex (dark). The buffer is 20 mM HEPES, 150 mM NaCl, 2 mM CaCl_2_, pH 7.4. kMRE corresponds to MRE × 10^3^. AC, adenylate cyclase catalytic domain; CaM, calmodulin; far-UV, far-ultraviolet; MRE, mean residual ellipticity; SR-CD, synchrotron radiation circular dichroism.
(PDF)

S3 Fig Peptide map of CaM. A sequence coverage map of CaM was determined after 2 min digestion with pepsin. Each blue bar represents a single CaM peptide. Only those peptides selected for HDX-MS data analysis after filtering the complete dataset are displayed. Linear sequence coverage of 87.9% was achieved. CaM, calmodulin; HDX-MS, hydrogen/deuterium exchange mass spectrometry.
(PDF)

S4 Fig HDX-MS behavior of CaM in the presence of H-helix peptide. Relative fractional exchange data were calculated at each point and plotted as a function of peptide position for CaM and CaM + H-helix. Each dot corresponds to the average of three independent replicates. The fractional uptake difference plot shows the differences in uptake calculated between CaM alone and in the presence of H-helix. Uptake plots of all peptides selected for final HDX-MS analysis are displayed in S11 Fig. The data used to generate the figure can be found in S4 Data. CaM, calmodulin; HDX-MS, hydrogen/deuterium exchange mass spectrometry.
(PDF)

S5 Fig HDX-MS behavior of CaM in the presence of P_MLCK_ peptide. Relative fractional exchange data were calculated at each point and plotted as a function of peptide position for CaM and CaM + P_MLCK_ peptide. Each dot corresponds to the average of three independent replicates. The fractional uptake difference plot shows the differences in uptake calculated between CaM alone and in the presence of P_MLCK_. Uptake plots of all peptides selected for final HDX-MS analysis are displayed in S12 Fig. The data used to generate the figure can be found in S5 Data. CaM, calmodulin; HDX-MS, hydrogen/deuterium exchange mass spectrometry; P_MLCK_, myosin light-chain kinase peptide.
(PDF)

S6 Fig HDX-MS behavior of CaM in the presence of full-length AC. Relative fractional exchange data were calculated at each point and plotted as a function of peptide position for CaM and CaM + full-length AC. Each dot corresponds to the average of three independent replicates. The fractional uptake difference plot shows the differences in uptake calculated between CaM alone and in the presence of AC. Uptake plots of all peptides selected for final HDX-MS analysis are displayed in S10 Fig. The data used to generate the figure can be found in S3 Data. AC, adenylate cyclase catalytic domain; CaM, calmodulin; HDX-MS, hydrogen/deuterium exchange mass spectrometry.
(PDF)

S7 Fig Small angle X-ray scattering of the AC:CaM complex. (A) Calculated scattering curve of the BUNCH model shown in panel (B) (blue line) and experimental data (black dots). (B) Typical model of AC-CaM complex obtained using the program BUNCH. The AC domain is shown in green while CaM is shown in red, interacting through its C-terminal domain (see text for details). AC, adenylate cyclase catalytic domain; CaM, calmodulin.
(PDF)

S8 Fig Intrinsic disorder prediction within the AC364 protein. AC HDX-MS results upon CaM binding, and regions of predicted disorder within the AC were mapped to the sequence of the protein. X-labeled residues correspond to the site of partner (CaM) binding. Areas of highest solvent protection in the AC correspond to those involved in partner binding and are flanked by regions of structural disorder. Gray areas correspond to those in which CaM does not induce changes in HDX-MS. For the complete nomenclature of secondary structure elements, please see the legend of S1 Fig. AC, adenylate cyclase catalytic domain; CaM, calmodulin; HDX-MS, hydrogen/deuterium exchange mass spectrometry.
(PDF)

S9 Fig Intrinsic disorder prediction within CaM. CaM HDX-MS results upon AC binding and those residues involved in protein/protein interactions were mapped to the sequence of the protein. Areas of highest solvent protection in the protein correspond to those involved in partner binding, as determined by X-ray Crystallography (X-labels). Gray areas correspond to those in which the AC does not induce changes in HDX-MS, or in which there is a lack of HDX-MS data. AC, adenylate cyclase catalytic domain; CaM, calmodulin; HDX-MS, hydrogen/deuterium exchange mass spectrometry.
(PDF)

S10 Fig HDX-MS results for individual AC peptides, both from AC in isolation and in complex with CaM. Black indicates the AC alone state, while red indicates AC in complex with CaM. AC, adenylate cyclase catalytic domain; CaM, calmodulin; HDX-MS, hydrogen/deuterium exchange mass spectrometry.
(PDF)

S11 Fig HDX-MS results for individual CaM peptides in complex with AC and H-helix peptide. For each peptide, CaM alone is colored black and CaM in the presence of H-helix is colored red, while CaM in the presence of AC is colored blue. AC, adenylate cyclase catalytic domain; CaM, calmodulin; HDX-MS, hydrogen/deuterium exchange mass spectrometry.
(PDF)

S12 Fig HDX-MS results for individual CaM peptides in complex with P_MLCK_. Black indicates the CaM alone state, while purple indicates CaM in complex with P_MLCK_. CaM, calmodulin; HDX-MS, hydrogen/deuterium exchange mass spectrometry; P_MLCK_, myosin light-chain kinase peptide.
(PDF)

S1 Table SAXS data collection and scattering derived parameters. ^a^ R_g_ obtained with the Guinier approximation in the range qR_g_ < 1.^b^ The calculated masses were derived from the sequences.^b1^ Corresponds to the 1H:1CaM complex.^b2^ Corresponds to the 2H:1CaM complex.^c^ Calculated from the sequence using the program sednterp, available at http://rasmb.org/sednterp/.^d^ Molecular mass M obtained from the whole I(q) curve (q_max_ = 0.4 Å^-1^) using the SAXS-MoW2 program, available at http://saxs.ifsc.usp.br/.^e^ Molecular mass M obtained from the whole I(q) curve (q_max_ = 0.2–0.25 Å^-1^) using the ScÅtter3 program, available at https://bl1231.als.lbl.gov/scatter/.
These two mass determinations do not depend on the value of the protein concentration, c. In general, they provide a useful complement to the derivation of M from the I(0)/c value but are especially relevant in cases such as CaM, which does not possess any tryptophan. CaM, calmodulin; q, momentum transfer; R_g_, radius of gyration; SAXS, small-angle X-ray scattering.
(PDF)

S2 Table Structural content of the isolated proteins and complexes derived from SR-CD data. The helical content from SR-CD was estimated using the BestSel software [12]. The helical content of AC364 (36%) in the AC:C-CaM complex [7] was computed using DSSP [13, 14]. AC, adenylate cyclase catalytic domain; C-CaM, C-terminal domain of CaM; DSSP, Dictionary of Secondary Structure of Proteins; SR-CD, synchrotron radiation circular dichroism.
(PDF)

S3 Table Estimation of the CaM-induced helical content increase in AC364 from SR-CD. The helical content increase (“difference in residues” column) provides an estimation of 41 amino acids that undergo a conversion from a disordered to a helical structure. AC364 and CaM contain 364 and 148 amino acids, respectively. CaM, calmodulin; SR-CD, synchrotron radiation circular dichroism.
(PDF)

S4 Table Estimation of the CaM-induced helical content increase in the H:CaM complex from SR-CD data. Assuming that the helical structure content does not change upon H:CaM complex formation, the number of amino acids in helical conformation would be 80 for a complex of 171 residues, (i.e., 76 + 4 residues from CaM and H-helix peptide, respectively; 47 ± 4% of helices in H:CaM complex). The helical content of the H:CaM complex estimated by BestSel from the SR-CD data is 60 ± 5, corresponding to 102 residues. Therefore, the H:CaM complex formation induces the conversion of approximately 22 residues in helical conformation. CaM, calmodulin; SR-CD, synchrotron radiation circular dichroism.
(PDF)

S1 Data (XLSX)

S2 Data (XLSX)

S3 Data (XLSX)

S4 Data (XLSX)

S5 Data (XLSX)

## Abbreviations

AC: adenylate cyclase catalytic domain
ACN: acetonitrile
AID: auto-inhibitory domain
CaM: calmodulin
CBS: CaM-binding site
C-CaM: C-terminal domain of CaM
CR1: catalytic region 1
CR2: catalytic region 2
CR3: catalytic region 3
CyaA: adenylate cyclase toxin
D_max_: maximal dimension
DSSP: Dictionary of Secondary Structure of Proteins
EOM: ensemble optimization method
far-UV: far-ultraviolet
FDR: false discovery rate
HDX-MS: hydrogen/deuterium exchange mass spectrometry
IDR: intrinsically disordered region
LHL: loop-helix-loop
MEMHDX: Mixed-Effects Model for HDX experiments
MLCK: myosin light chain kinase
MoRF: molecular recognition feature
MRE: mean residual ellipticity
MS: mass spectrometry
MS^E^: data-independent acquisition scheme
N-CaM: N-terminal domain of CaM
NSD: normalized spatial discrepancy
PA: protective antigen
pdb: Protein Data Bank
PLGS: Protein Lynx Global Server
P_MLCK_: myosin light-chain kinase peptide
R_g_: radius of gyration
SASBDB: Small Angle Scattering Biological Data Bank
SAXS: small-angle X-ray scattering
SEC: size exclusion chromatography
SLIM: short linear motif
SR-CD: synchrotron radiation circular dichroism
T18: C-terminal trypsin-cleavage fragment of CyaA (amino acids 225–364)
T18b1: first beta-sheet of the T18 fragment
T18b2: second beta-sheet of the T18 fragment
T1SS: type I secretion system
T25: N-terminal trypsin-cleavage fragment of CyaA (amino acids 1–224)
