# Supplementary figures and images for: Calmodulin fishing with a structurally disordered bait triggers CyaA catalysis
Source: PLoS Biol. 2017 Dec 29;15(12):e2004486. doi: 10.1371/journal.pbio.2004486 (PMC5764468; doi:10.1371/journal.pbio.2004486)

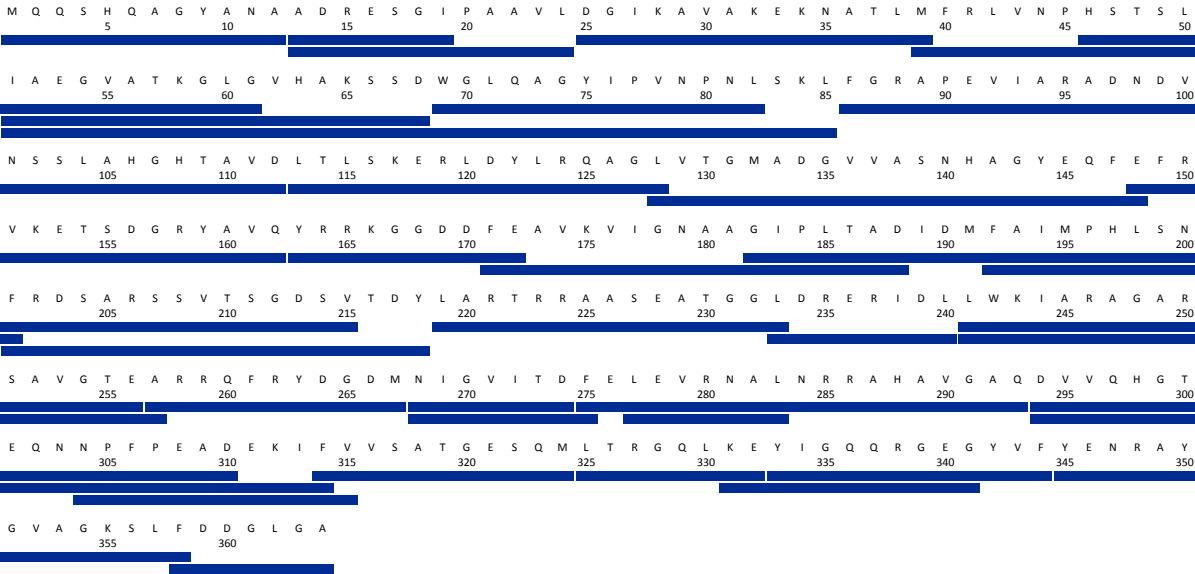

Total: 36 Peptides, 100.0% Coverage, 1.46 Redundancy

Supplement: S1 Fig — A sequence coverage map of AC was determined after 2 min digestion with pepsin. Each blue bar represents a single AC peptide. Only those peptides selected for HDX-MS data analysis after filtering the complete dataset are displayed. Linear sequence coverage of 100% was achieved. The secondary structure architecture of AC is as follows: A-helix (residues 11–17), B-helix (residues 20–34), C-helix (residues 44–53), D-helix (residues 89–106), E-helix (residues 116–126), F-helix (198–210), G-helix (214–223), Hom-loop (residues 226–232), H-helix (residues 234–253), H′-helix (residues 255–259), T18b1 (residues 262–270), I-helix (residues 274–289), catalyic loop (residues 300–312), T18b2 (residues 313–325), J-helix (residues 326–340), and an LHL motif (residues 341–364). The catalytic site is made of three highly conserved regions that are directly involved in substrate binding and catalysis: CR1 (residues 54–77), CR2 (residues 184–198), and CR3 (residues 295–315). AC, catalytic domain; CR1, catalytic region 1; CR2, catalytic region 2; CR3, catalytic region 3; LHL, loop-helix-loop; T18b1, first beta-sheet of the T18 fragment; T18b2, second beta-sheet of the T18 fragment. (PDF) [file pbio.2004486.s001.pdf]

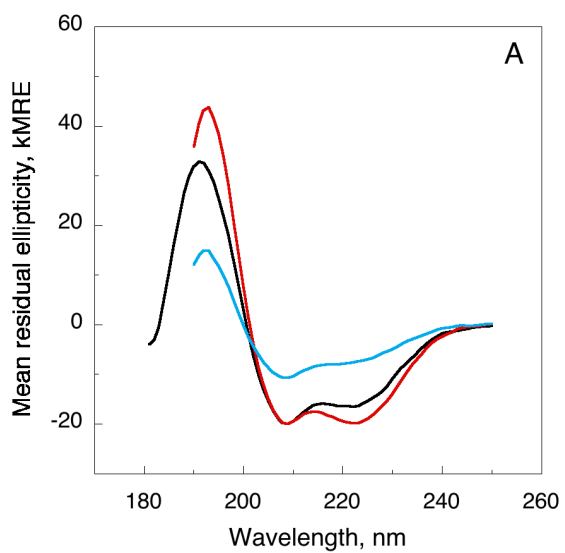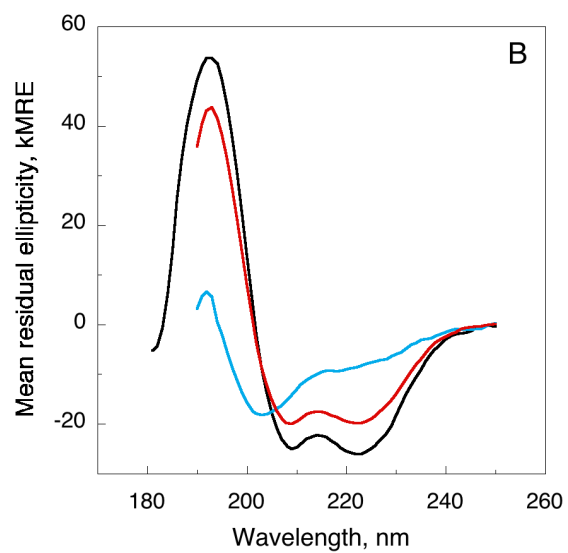

Supplement: S2 Fig — (A) AC (blue), CaM (red), and AC:CaM complex (dark). (B) H-helix peptide (blue), CaM (red), and H-helix:CaM complex (dark). The buffer is 20 mM HEPES, 150 mM NaCl, 2 mM CaCl2, pH 7.4. kMRE corresponds to MRE × 103. AC, adenylate cyclase catalytic domain; CaM, calmodulin; far-UV, far-ultraviolet; MRE, mean residual ellipticity; SR-CD, synchrotron radiation circular dichroism. (PDF) [file pbio.2004486.s002.pdf]

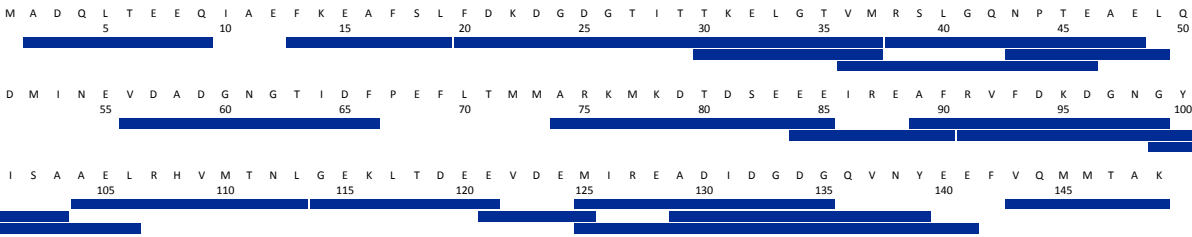

Total: 20 Peptides, 87.9% Coverage, 1.53 Redundancy

Supplement: S3 Fig — A sequence coverage map of CaM was determined after 2 min digestion with pepsin. Each blue bar represents a single CaM peptide. Only those peptides selected for HDX-MS data analysis after filtering the complete dataset are displayed. Linear sequence coverage of 87.9% was achieved. CaM, calmodulin; HDX-MS, hydrogen/deuterium exchange mass spectrometry. (PDF) [file pbio.2004486.s003.pdf]

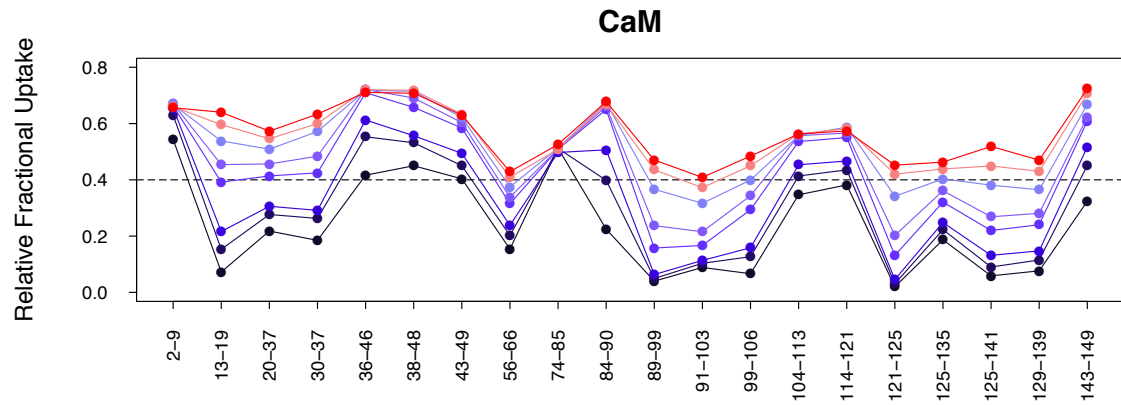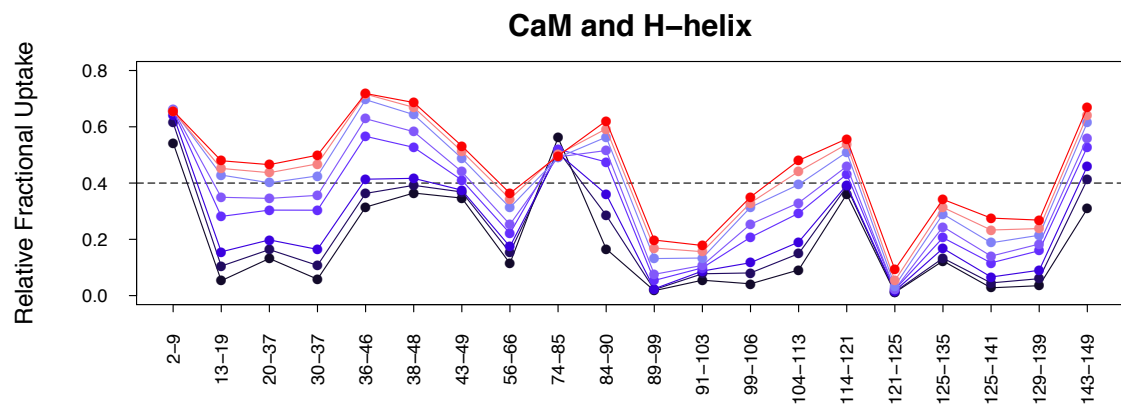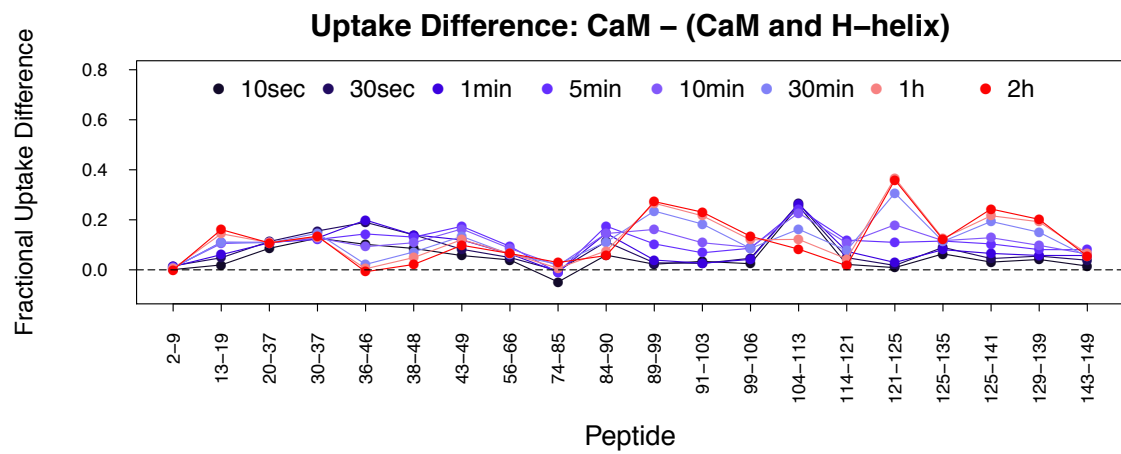

Supplement: S4 Fig — Relative fractional exchange data were calculated at each point and plotted as a function of peptide position for CaM and CaM + H-helix. Each dot corresponds to the average of three independent replicates. The fractional uptake difference plot shows the differences in uptake calculated between CaM alone and in the presence of H-helix. Uptake plots of all peptides selected for final HDX-MS analysis are displayed in S11 Fig. The data used to generate the figure can be found in S4 Data. CaM, calmodulin; HDX-MS, hydrogen/deuterium exchange mass spectrometry. (PDF) [file pbio.2004486.s004.pdf]

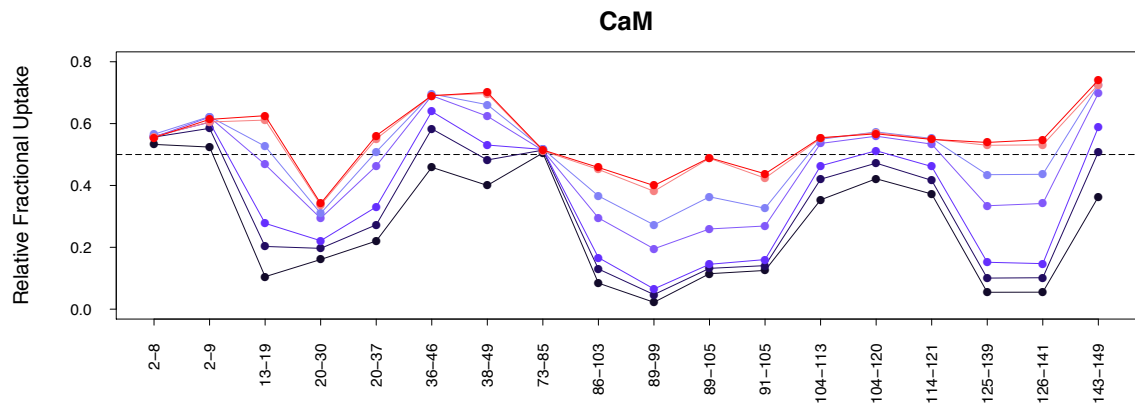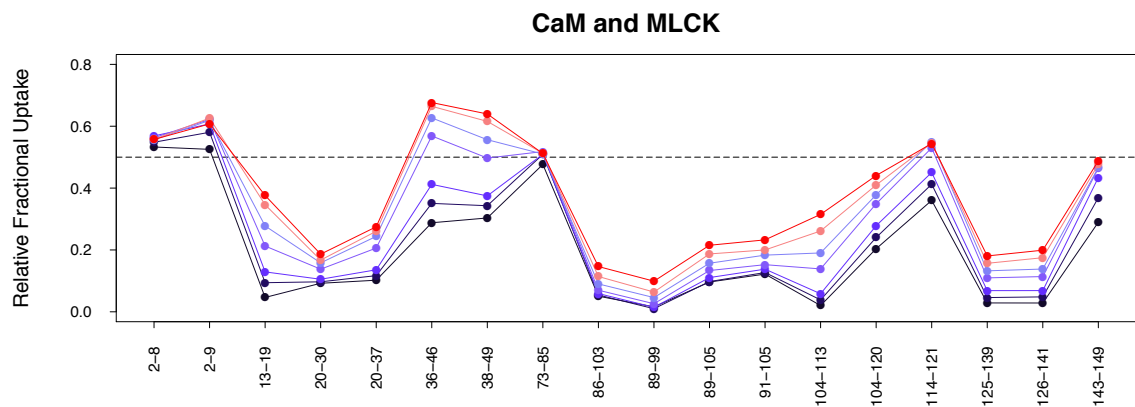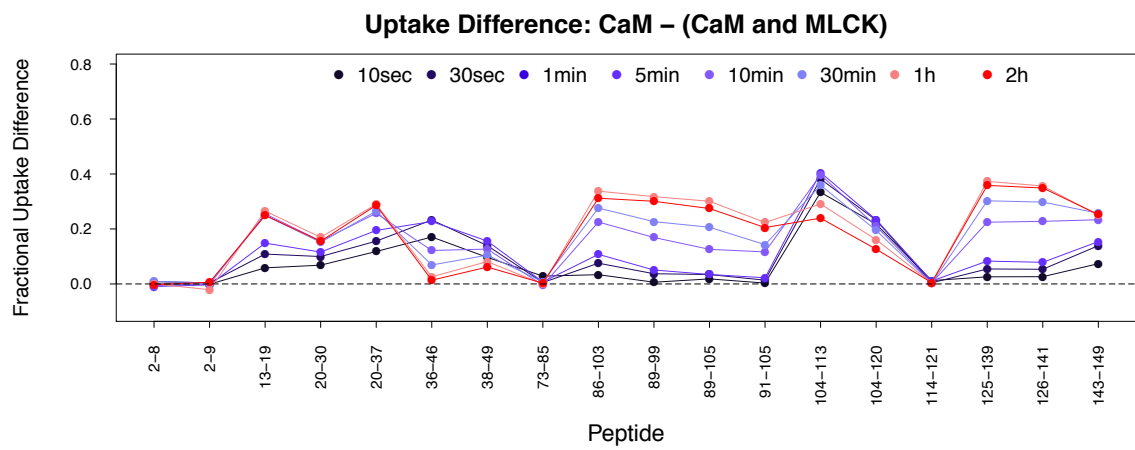

Supplement: S5 Fig — Relative fractional exchange data were calculated at each point and plotted as a function of peptide position for CaM and CaM + PMLCK peptide. Each dot corresponds to the average of three independent replicates. The fractional uptake difference plot shows the differences in uptake calculated between CaM alone and in the presence of PMLCK. Uptake plots of all peptides selected for final HDX-MS analysis are displayed in S12 Fig. The data used to generate the figure can be found in S5 Data. CaM, calmodulin; HDX-MS, hydrogen/deuterium exchange mass spectrometry; PMLCK, myosin light-chain kinase peptide. (PDF) [file pbio.2004486.s005.pdf]

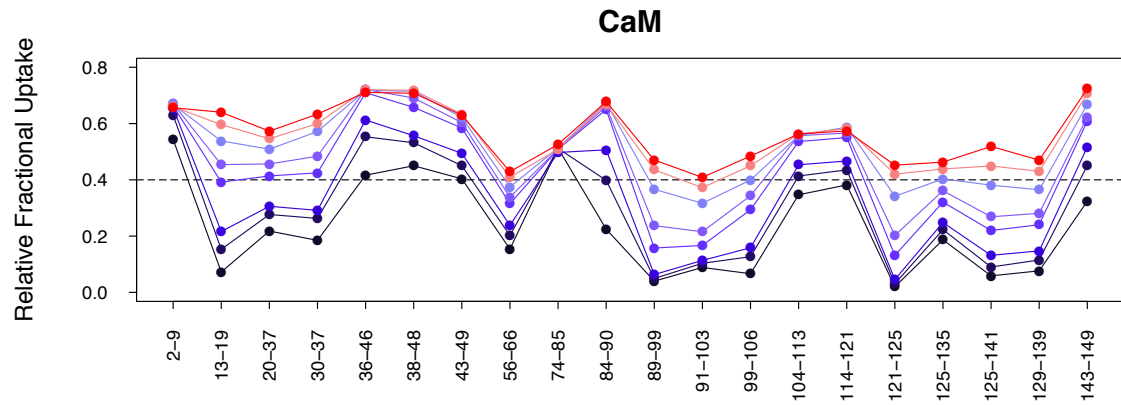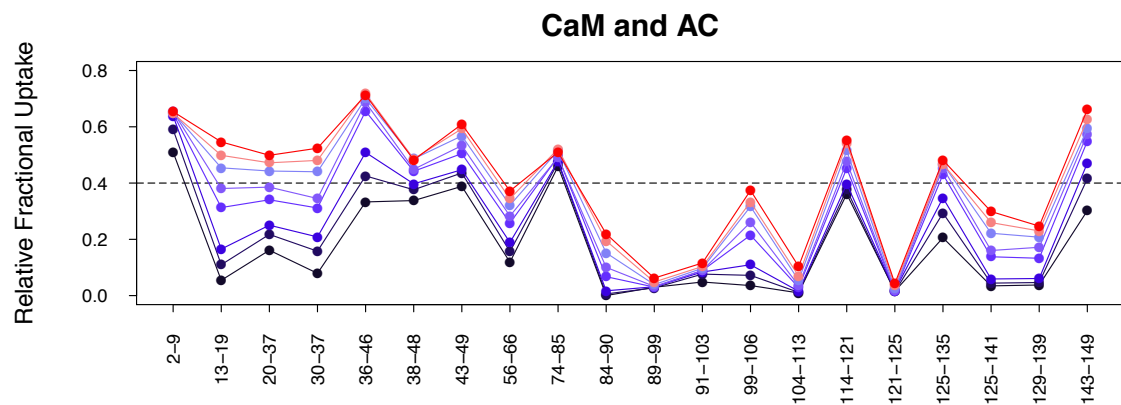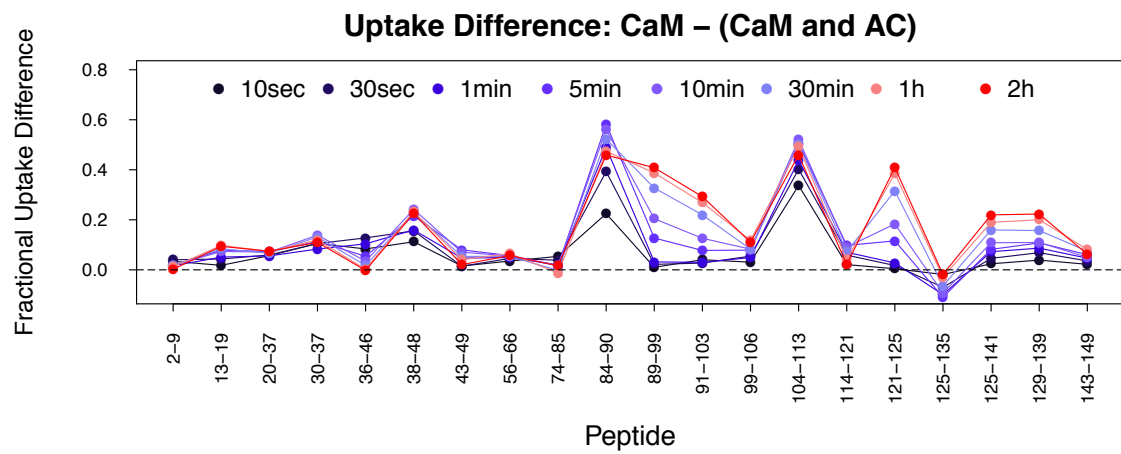

Supplement: S6 Fig — Relative fractional exchange data were calculated at each point and plotted as a function of peptide position for CaM and CaM + full-length AC. Each dot corresponds to the average of three independent replicates. The fractional uptake difference plot shows the differences in uptake calculated between CaM alone and in the presence of AC. Uptake plots of all peptides selected for final HDX-MS analysis are displayed in S10 Fig. The data used to generate the figure can be found in S3 Data. AC, adenylate cyclase catalytic domain; CaM, calmodulin; HDX-MS, hydrogen/deuterium exchange mass spectrometry. (PDF) [file pbio.2004486.s006.pdf]

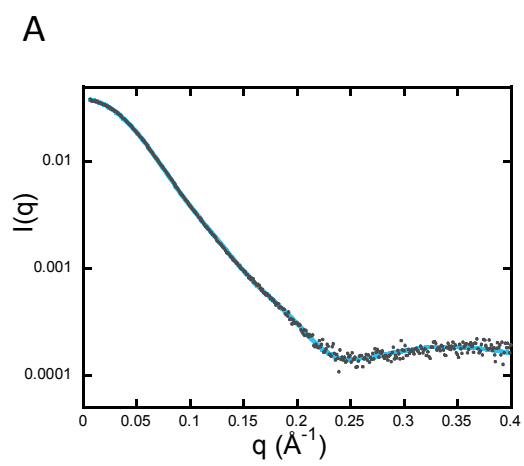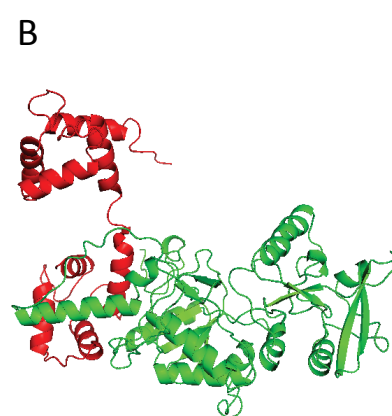

Supplement: S7 Fig — (A) Calculated scattering curve of the BUNCH model shown in panel (B) (blue line) and experimental data (black dots). (B) Typical model of AC-CaM complex obtained using the program BUNCH. The AC domain is shown in green while CaM is shown in red, interacting through its C-terminal domain (see text for details). AC, adenylate cyclase catalytic domain; CaM, calmodulin. (PDF) [file pbio.2004486.s007.pdf]

## CyaA CATALYTIC DOMAIN

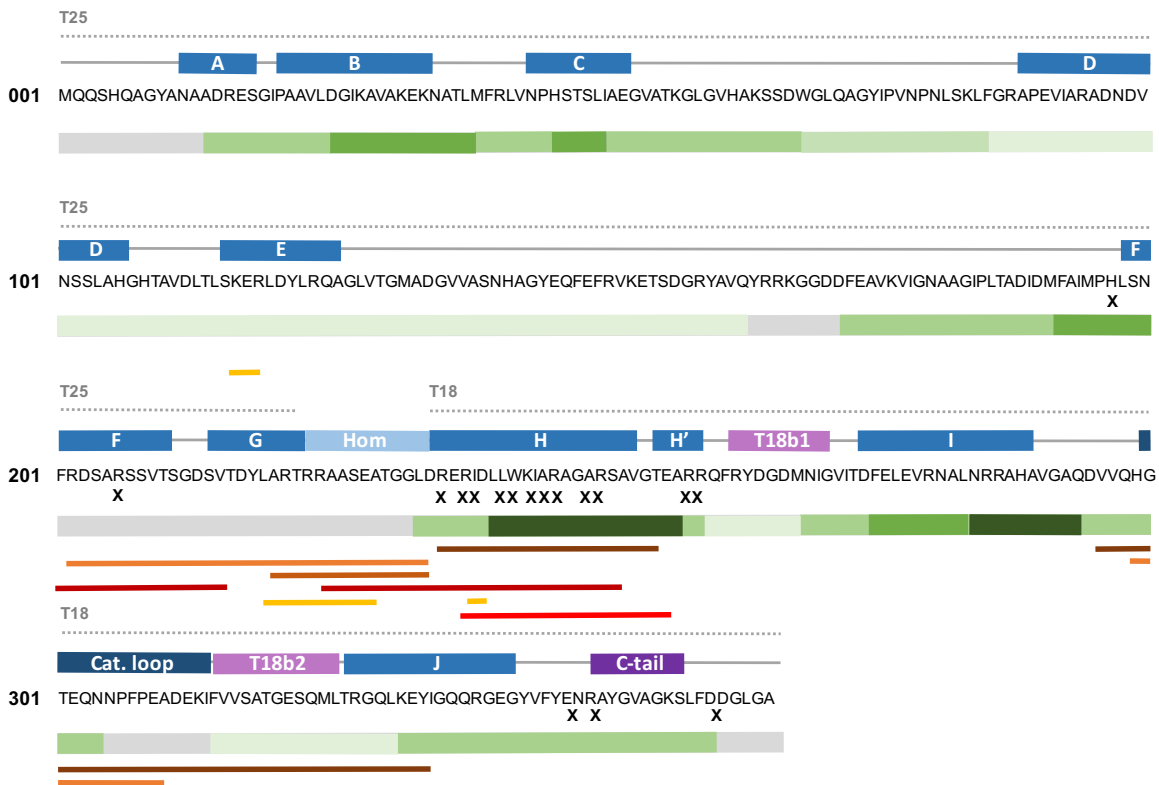

Supplement: S8 Fig — AC HDX-MS results upon CaM binding, and regions of predicted disorder within the AC were mapped to the sequence of the protein. X-labeled residues correspond to the site of partner (CaM) binding. Areas of highest solvent protection in the AC correspond to those involved in partner binding and are flanked by regions of structural disorder. Gray areas correspond to those in which CaM does not induce changes in HDX-MS. For the complete nomenclature of secondary structure elements, please see the legend of S1 Fig. AC, adenylate cyclase catalytic domain; CaM, calmodulin; HDX-MS, hydrogen/deuterium exchange mass spectrometry. (PDF) [file pbio.2004486.s008.pdf]

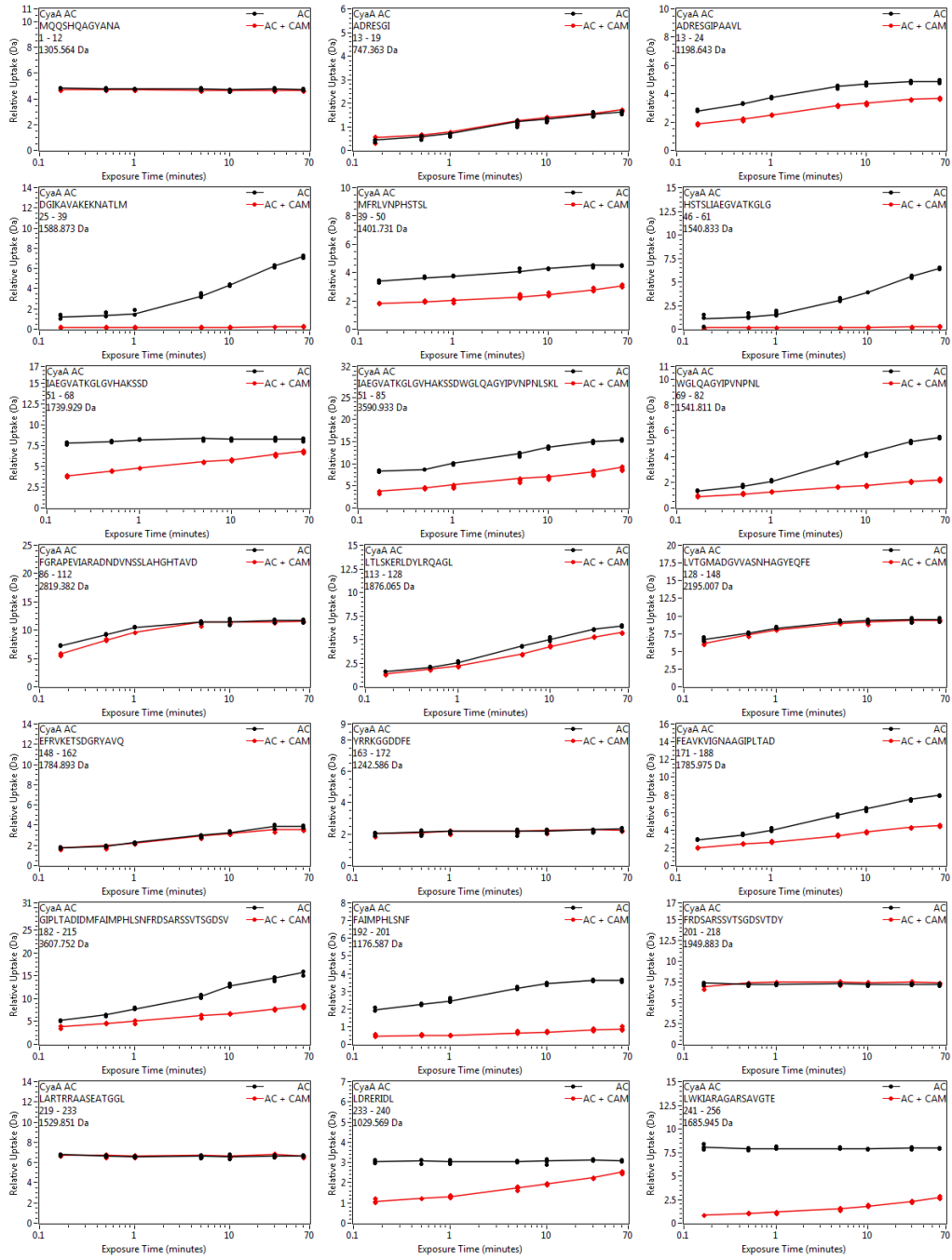

=>

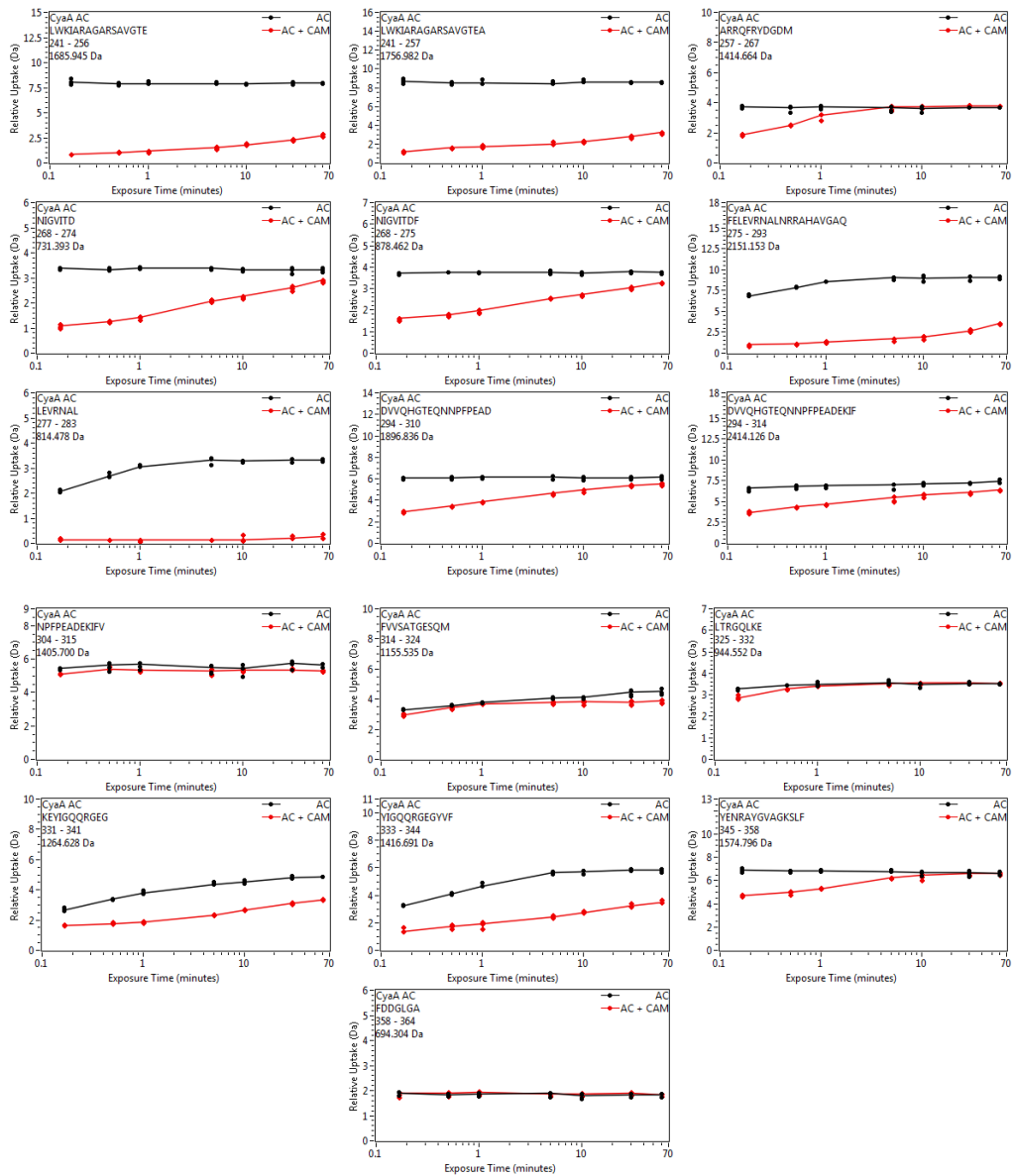

Supplement: S10 Fig — Black indicates the AC alone state, while red indicates AC in complex with CaM. AC, adenylate cyclase catalytic domain; CaM, calmodulin; HDX-MS, hydrogen/deuterium exchange mass spectrometry. (PDF) [file pbio.2004486.s010.pdf]

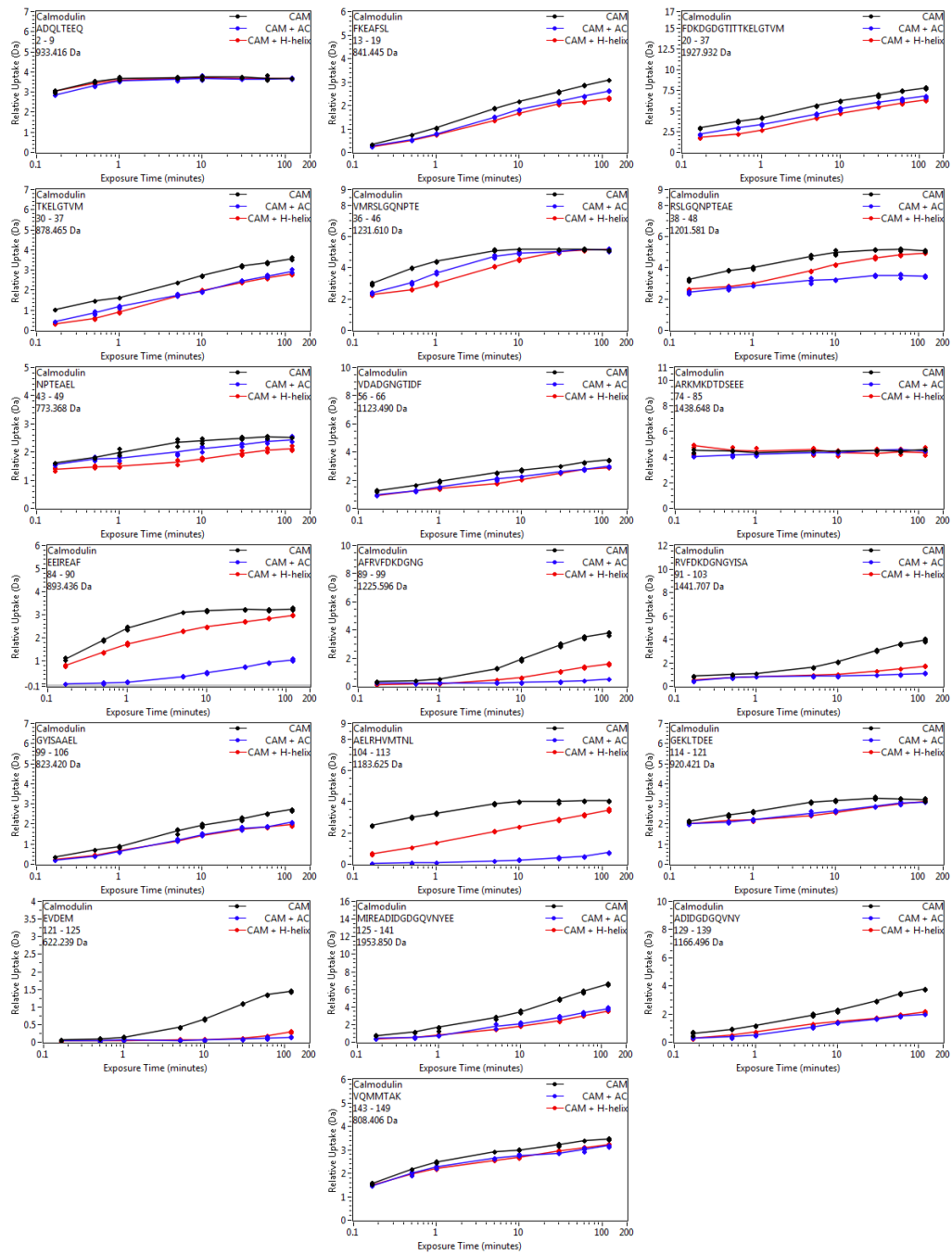

Supplement: S11 Fig — For each peptide, CaM alone is colored black and CaM in the presence of H-helix is colored red, while CaM in the presence of AC is colored blue. AC, adenylate cyclase catalytic domain; CaM, calmodulin; HDX-MS, hydrogen/deuterium exchange mass spectrometry. (PDF) [file pbio.2004486.s011.pdf]

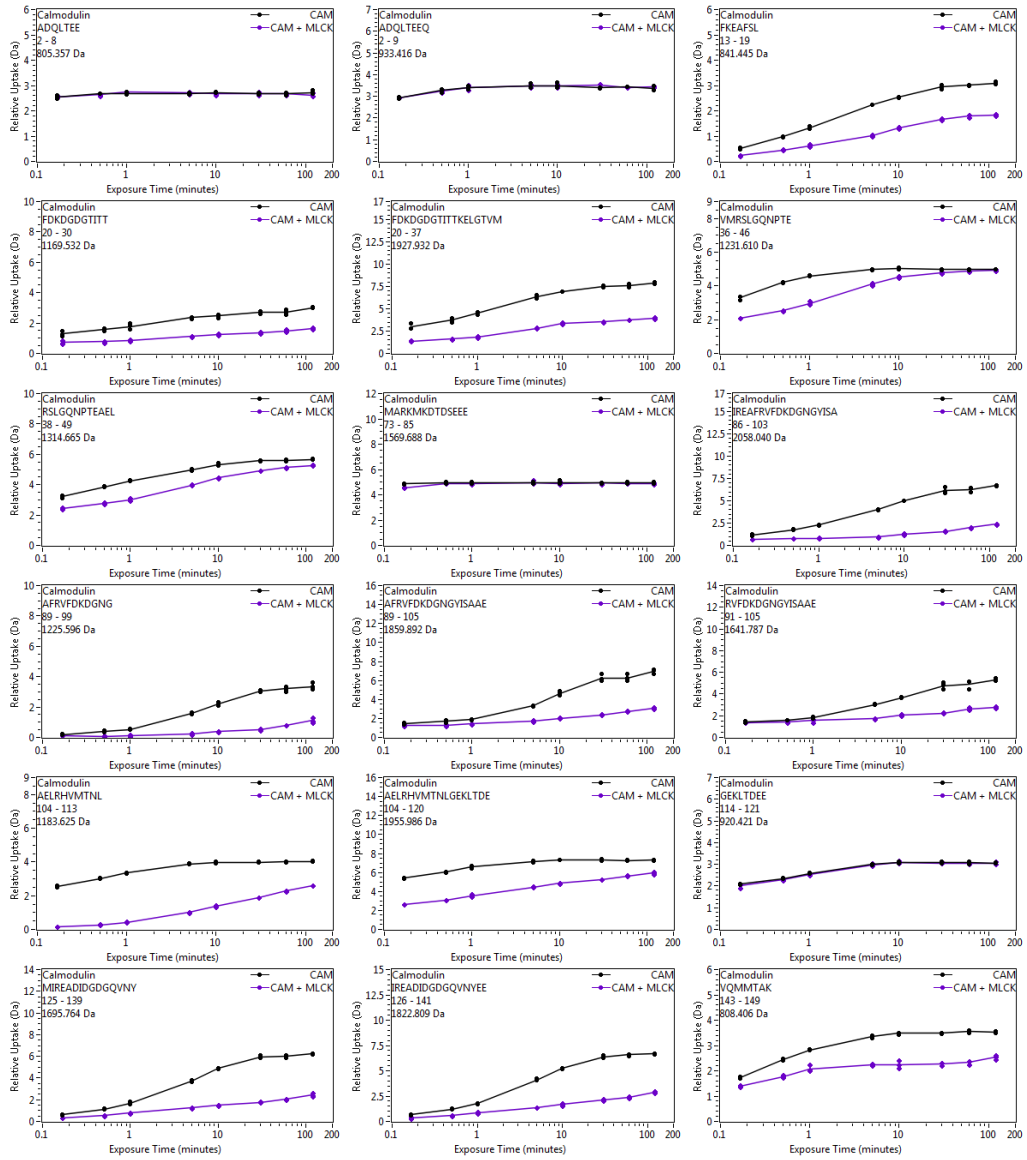

Supplement: S12 Fig — Black indicates the CaM alone state, while purple indicates CaM in complex with PMLCK. CaM, calmodulin; HDX-MS, hydrogen/deuterium exchange mass spectrometry; PMLCK, myosin light-chain kinase peptide. (PDF) [file pbio.2004486.s012.pdf]
