# Supplementary material for: Calmodulin fishing with a structurally disordered bait triggers CyaA catalysis
Source: PLoS Biol. 2017 Dec 29;15(12):e2004486. doi: 10.1371/journal.pbio.2004486 (PMC5764468; doi:10.1371/journal.pbio.2004486)
Supplement: S9 Fig — CaM HDX-MS results upon AC binding and those residues involved in protein/protein interactions were mapped to the sequence of the protein. Areas of highest solvent protection in the protein correspond to those involved in partner binding, as determined by X-ray Crystallography (X-labels). Gray areas correspond to those in which the AC does not induce changes in HDX-MS, or in which there is a lack of HDX-MS data. AC, adenylate cyclase catalytic domain; CaM, calmodulin; HDX-MS, hydrogen/deuterium exchange mass spectrometry. (PDF) [file pbio.2004486.s009.pdf]

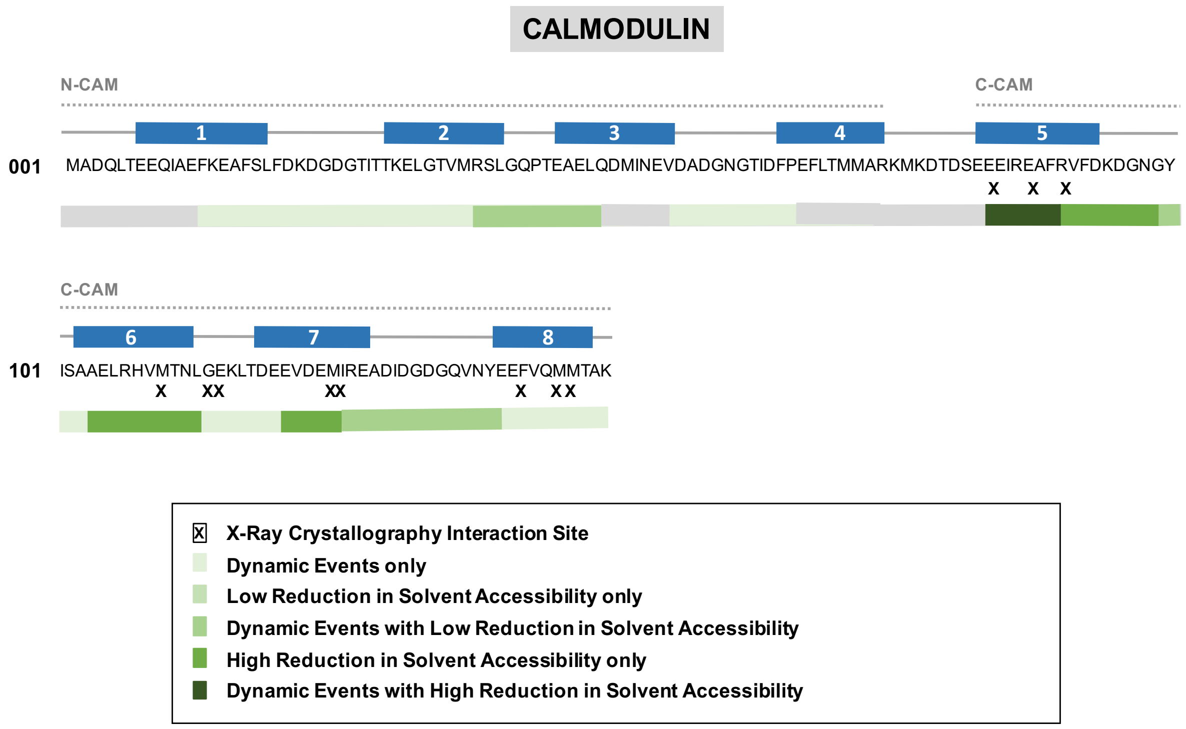

CALMODULIN
N-CAM
C-CAM
1
2
3
4
5
001
MADQLTEEQIAEFKEAFSLFDKDGDGTITTKELGTVMRSLGQPTEAELQDMINEVDADGNGTIDFPEFLTMMARKMKDTDSEEEIREAFRVFDKDGNGY
X X X
C-CAM
6
7
8
101
ISAAELRHVMTNLGEKLTDEEVDEMIREADIDGDGQVNYEEFVQMMTAK
X XX XX X XX
X-Ray Crystallography Interaction Site
Dynamic Events only
Low Reduction in Solvent Accessibility only
Dynamic Events with Low Reduction in Solvent Accessibility
High Reduction in Solvent Accessibility only
Dynamic Events with High Reduction in Solvent Accessibility
