# Supplementary material for: Calmodulin fishing with a structurally disordered bait triggers CyaA catalysis
Source: PLoS Biol. 2017 Dec 29;15(12):e2004486. doi: 10.1371/journal.pbio.2004486 (PMC5764468; doi:10.1371/journal.pbio.2004486)
Supplement: S1 Table — a Rg obtained with the Guinier approximation in the range qRg < 1.b The calculated masses were derived from the sequences.b1 Corresponds to the 1H:1CaM complex.b2 Corresponds to the 2H:1CaM complex.c Calculated from the sequence using the program sednterp, available at http://rasmb.org/sednterp/.d Molecular mass M obtained from the whole I(q) curve (qmax = 0.4 Å-1) using the SAXS-MoW2 program, available at http://saxs.ifsc.usp.br/.e Molecular mass M obtained from the whole I(q) curve (qmax = 0.2–0.25 Å-1) using the ScÅtter3 program, available at https://bl1231.als.lbl.gov/scatter/. These two mass determinations do not depend on the value of the protein concentration, c. In general, they provide a useful complement to the derivation of M from the I(0)/c value but are especially relevant in cases such as CaM, which does not possess any tryptophan. CaM, calmodulin; q, momentum transfer; Rg, radius of gyration; SAXS, small-angle X-ray scattering. (PDF) [file pbio.2004486.s013.pdf]

	**CaM**	**H-CaM**	**P_{MLCK}-CaM**	**AC364**	**AC364-CaM**
**Data collection parameters**					
Instrument	Beamline SWING (synchrotron SOLEIL)				
Detector	CCD-based AVIEX				
Beam geometry	0.8 mm x 0.15 mm				
Wavelength [Å]	1.0				
q-range [$Å^{-1}$]	$0.0064 < q < 0.50$				
Exposure time [s]	2				
Temperature [K]	288				
**Structural parameters**					
I(0) Guinier [cm^{-1}]	0.0317	0.0159	0.0134	0.0252	0.0381
R_g Guinier [Å] [a]	22.2	21.9	17.4	25.9	29.2
I(0) p(r) [cm^{-1}]	0.0319	0.0159	0.0134	0.0252	0.0383
R_g p(r) [Å]	22.7	22.1	17.3	26.2	29.7
D_{Max} [Å]	78	75	52	91	98
**Molecular mass determination**					
$MM_{sequence}$ [kDa] [b]	16.8	19.3 [b1]-21.7 [b2]	19.0	39.4	56.2
Partial specific volume [$cm^3.g^{-1}$] [c]	0.7174	0.7210	0.7191	0.7233	0.7215
Concentration [$g.L^{-1}$]	ND*	ND*	ND*	0.80	0.82
$MM_{I(0)/c}$ [kDa]	ND*	ND*	ND*	38.6	56.3
$MM_{SAXS\ MoW}$ [kDa] [d]	17.9	21.9	18.6	41.9	58.3
$MM_{SAXS\ QR}$ [kDa] [e]	16.8	19.7	19.2	40.2	54.3
**Dammif/Dammin analysis**					
Dammif Model number	20	20	20		
NSD	0.56	0.53	0.46		
c^2 (Dammin)	1.56	1.10	1.90		
