# Supplementary material for: Calmodulin fishing with a structurally disordered bait triggers CyaA catalysis
Source: PLoS Biol. 2017 Dec 29;15(12):e2004486. doi: 10.1371/journal.pbio.2004486 (PMC5764468; doi:10.1371/journal.pbio.2004486)
Supplement: S2 Table — The helical content from SR-CD was estimated using the BestSel software [12]. The helical content of AC364 (36%) in the AC:C-CaM complex [7] was computed using DSSP [13, 14]. AC, adenylate cyclase catalytic domain; C-CaM, C-terminal domain of CaM; DSSP, Dictionary of Secondary Structure of Proteins; SR-CD, synchrotron radiation circular dichroism. (PDF) [file pbio.2004486.s014.pdf]

Sample	SR-CD
	Helical Content, % ± S.D.
holo-AC alone	27 ± 2
holo-CaM	51 ± 3
holo-AC:CaM	42 ± 3
holo-H	19 ± 1
holo-H:CaM	60 ± 5
