# Supplementary material for: Calmodulin fishing with a structurally disordered bait triggers CyaA catalysis
Source: PLoS Biol. 2017 Dec 29;15(12):e2004486. doi: 10.1371/journal.pbio.2004486 (PMC5764468; doi:10.1371/journal.pbio.2004486)
Supplement: S3 Table — The helical content increase (“difference in residues” column) provides an estimation of 41 amino acids that undergo a conversion from a disordered to a helical structure. AC364 and CaM contain 364 and 148 amino acids, respectively. CaM, calmodulin; SR-CD, synchrotron radiation circular dichroism. (PDF) [file pbio.2004486.s015.pdf]

Sample	SR-CD	
	**Helical Content, %**	**Residues**
1 AC	27	98
2 CaM	51	76
3 AC + CaM*	34	174
4 AC: CaM complex	42	215
Difference 4 - 3	8	41

* average of the two previous values weighted by the respective residue number.
