# Supplementary material for: Calmodulin fishing with a structurally disordered bait triggers CyaA catalysis
Source: PLoS Biol. 2017 Dec 29;15(12):e2004486. doi: 10.1371/journal.pbio.2004486 (PMC5764468; doi:10.1371/journal.pbio.2004486)
Supplement: S4 Table — Assuming that the helical structure content does not change upon H:CaM complex formation, the number of amino acids in helical conformation would be 80 for a complex of 171 residues, (i.e., 76 + 4 residues from CaM and H-helix peptide, respectively; 47 ± 4% of helices in H:CaM complex). The helical content of the H:CaM complex estimated by BestSel from the SR-CD data is 60 ± 5, corresponding to 102 residues. Therefore, the H:CaM complex formation induces the conversion of approximately 22 residues in helical conformation. CaM, calmodulin; SR-CD, synchrotron radiation circular dichroism. (PDF) [file pbio.2004486.s016.pdf]

Sample	Helical Content, % ± S.D.	Total no. of residues	No. of AA in helices
CaM	51 ± 3	148	76
H-helix peptide	19 ± 1	22	4
Sum of H:CaM assuming no helical structure changes	47 ± 4	171	80
H-helix:CaM	60 ± 5	171	102
